# Global mpox outbreak dynamics during and after the 2024–2026 public health emergency

**DOI:** 10.1371/journal.pone.0350572

**Published:** 2026-10-21

**Authors:** Tambe Elvis Akem

**Affiliations:** Independent Public Health Consultant, Yaounde, Cameroon; Virginia Commonwealth University, UNITED STATES OF AMERICA

## Abstract

The second WHO Public Health Emergency of International Concern (PHEIC) for mpox was declared on 14 August 2024 after clade Ib monkeypox virus emerged in the Democratic Republic of the Congo (DRC) and spread rapidly across Africa and internationally. The PHEIC ended on 5 September 2025, and the Africa CDC Public Health Emergency of Continental Security was lifted on 22 January 2026. This study characterised global and Africa-specific mpox epidemiology across the full second PHEIC cycle and immediate post-emergency period, from January 2024 to February 2026. A retrospective surveillance analysis was conducted using the WHO Global Mpox Trends dashboard exported data, supplemented by WHO situation reports S32–S64 and international and national surveillance reports. Analyses included negative binomial segmented regression, travel-linked pathway analysis, exploratory analysis of documented secondary transmission after travel-linked detection, and descriptive statistics. Models used reported case counts with log population offsets; reported case-rate ratios therefore reflect reported case rates rather than true infection incidence. Between January 2024 and February 2026, 82,738 confirmed cases and 306 deaths were reported to WHO globally (case fatality ratio 0.37%). The PHEIC period accounted for 74.0% of cases, peaking at 7,405 cases in May 2025. In total, 163 clade-specific importation or travel-linked detection events were documented across 41 countries within the primary analytic cutoff; documented secondary transmission occurred in 25 (15.3%) events across 16 countries. Within DRC, 200 health zones recorded 144,035 total reported cases and 2,047 deaths (case fatality ratio 1.42%); these aggregate suspected/clinically compatible and confirmed cases. The top 20 health zones accounted for 45.4% of national burden. Reported transmission declined after the May 2025 peak but persisted beyond PHEIC termination. Emergency termination marked an administrative transition, not the end of mpox transmission. The post-emergency period requires sustained surveillance, maintained diagnostic capacity, targeted response in persistent transmission settings, and equitable access to vaccines and countermeasures.

## Introduction

Mpox, caused by monkeypox virus (MPXV), is a zoonotic orthopoxviral disease [1]. The disease was first described in humans in the Democratic Republic of the Congo (DRC) in 1970 [2]. Two main phylogenetic clades are recognised, clade I and clade II [1], and the 2022–2023 multi-country outbreak was driven largely by clade IIb, with transmission concentrated predominantly among gay, bisexual and other men who have sex with men (MSM) in non-endemic settings [3]. Children and adolescents represented a small minority of reported cases globally during this period [4].

WHO declared its first mpox public health emergency of international concern (PHEIC) in July 2022 [5]. Under the International Health Regulations (2005), a PHEIC is a formal mechanism to signal that an extraordinary public health event may require coordinated international action; it is a governance and coordination instrument. The first PHEIC ended in May 2023 after sustained global decline [6]. In 2023 and 2024, clade Ib outbreaks in eastern DRC, initially in South Kivu, were characterised by sustained human-to-human transmission and increasing regional spread [7–9].

The 2024–2026 emergency cycle unfolded in a multi-clade epidemiological context. Clade IIb transmission persisted after the 2022–2023 global outbreak, including in South-East Asian and Western Pacific countries where mpox activity had already been documented before the second PHEIC [10,11]. Clade Ia remained important in historically affected Central African settings, while clade Ib emerged in eastern DRC and spread regionally and internationally [6,8,9]. Although all presentations are classified as mpox, clade attribution remains important for surveillance, severity interpretation, and response planning.

Africa CDC declared a Public Health Emergency of Continental Security (PHECS) on 13 August 2024 [12], followed by WHO’s second mpox PHEIC on 14 August 2024 [13], triggering intensified international response efforts [14]. WHO terminated the PHEIC on 5 September 2025 [15], and Africa CDC lifted the PHECS on 22 January 2026 [16]. This study integrates global and African surveillance trends, DRC sub-national burden, and cross-border spread across the full second emergency cycle and post-emergency transition to inform post-PHEIC planning.

## Methods

### Data sources and study period

A retrospective surveillance analysis was carried out. The primary analytic database was extracted and validated from the WHO Global Mpox Trends dashboard [17], which provided the core time-series data for this study: global and regional monthly reported confirmed case counts, African weekly surveillance, DRC sub-national health-zone burden, demographic and epidemiological characteristics, and international importation events. For age-sex and the epidemiological variables MSM, persons living with HIV (PLHIV), health worker status, travel history, sexual transmission, hospitalization, intensive care unit (ICU) admission, and death, estimates were calculated using all cases with available data for each variable from 2022 onward; the recent period reflected the most recent 12-month dashboard window available at extraction, ending in March 2026. WHO multi-country mpox external situation reports (sitreps S32-S64) [18] were used to validate selected dashboard-derived data points, construct the policy-response timeline, and reconcile discrepancies across sources. Additional triangulation drew on Africa CDC epidemiological updates [19], US CDC surveillance summaries [20], European Centre for Disease Prevention and Control (ECDC) surveillance data [21], and national public health reports. Population denominators for regression offsets were obtained from the United Nations Population Division portal [22]. The primary study period covered January 2024–28 February 2026; selected descriptive analyses incorporated data available through March 2026, where indicated.

All WHO Global Mpox Trends dashboard datasets were downloaded in April 2026. Exact data cutoffs varied by analytical component: global monthly confirmed case analyses and importation/travel-linked detection analyses used the 28 February 2026 primary analytic cutoff; African weekly surveillance data were current through 22 March 2026, but for the primary interrupted time-series analysis weekly confirmed case counts were truncated to the 28 February 2026 analytic cutoff; DRC subnational health-zone burden used data current to mid-March 2026; and demographic and epidemiological characteristic data were available through March 2026. The dashboard data reflect information reported by national public health authorities and are subject to retrospective revision, differential reporting completeness, variable testing capacity, and differing case definitions across countries and time periods. WHO region assignments follow the standard six-region classification. Reporting followed STROBE guidelines, and the completed checklist is provided as S1 Checklist.

### Phase definitions

Three epidemic phases were defined based on official declarations: Pre-PHEIC (1 January to 13 August 2024); PHEIC (14 August 2024–5 September 2025); and Post-PHEIC (6 September 2025–28 February 2026). For monthly analyses, transition months were assigned to the preceding phase. A post-PHEIC sub-phase analysis distinguished PHECS (6 September 2025–22 January 2026) and post-PHECS (23 January to 28 February 2026) periods.

### Statistical analysis

Segmented regression models. Monthly global and regional case counts were modelled using negative binomial (NB) segmented regression with log-population offsets. Model selection between Poisson and NB models was guided by overdispersion diagnostics, including comparison of Pearson chi-square dispersion under Poisson models and estimation of the NB dispersion parameter alpha. NB models were retained where the data showed overdispersion relative to Poisson assumptions. Residual autocorrelation was assessed using the Durbin-Watson statistic on Pearson residuals. Residual autocorrelation was detected in some model residuals and is noted as a diagnostic finding; the primary analysis was conducted using the default covariance estimator without HAC (heteroskedasticity and autocorrelation consistent) correction, as the primary models did not apply HAC standard errors. African weekly confirmed case counts were analysed using the same NB segmented regression framework, with log-population offsets based on corresponding population denominators. Suspected case counts were not combined with confirmed case counts in regression models. Two breakpoints were prespecified on substantive grounds prior to analysis: the WHO PHEIC declaration on 14 August 2024, representing the formal onset of the emergency period and its associated intensification of surveillance and response; and the April 2025 WHO Strategic Preparedness and Response Plan (SPRP) update, representing the major mid-emergency policy revision. These breakpoints were not selected to fit the data and were not moved after inspection of model results. Because the April 2025 breakpoint falls close to the May 2025 global case peak, the subsequent decline identified in the model largely reflects the natural post-peak trajectory of the epidemic rather than any effect of the SPRP update; this confounding with the natural epidemic inflection is addressed in the Results and Discussion. For sensitivity analysis, the April 2025 breakpoint was shifted by minus one, plus one, and plus two months to assess whether the direction and approximate magnitude of model estimates were robust to the exact breakpoint location. Model outputs are expressed as reported case-rate ratios (CRRs), defined as ratios of reported case rates between periods or trend segments, derived from NB coefficients with log-population offsets. CRRs reflect reported case rates, not true infection incidence, and therefore capture both transmission dynamics and changes in surveillance intensity.

Travel-linked detection and secondary-transmission analysis. The 163 WHO dashboard importation or travel-linked detection records within the primary analytic cutoff were classified by clade, reported place of origin, destination WHO region, epidemic phase, and secondary transmission status. Secondary transmission (ST) was defined as a binary indicator of any documented onward case or cases following a travel-linked detection event. This definition captures events with a single secondary case as well as those with multiple secondary cases, and does not distinguish isolated secondary transmission from sustained local spread. ST was treated as a documented surveillance outcome, not as a measure of transmissibility or sustained community transmission. The analysis did not capture ongoing clade IIb transmission reported through routine national surveillance. A directed country-to-country travel-pathway network was constructed using harmonised country labels. In this network, place of origin represents reported travel history and does not confirm the site of infection or transmission. An exploratory logistic regression model examined binary ST status as the outcome; destination region and origin group were the final predictors to limit overfitting given only 25 ST events. This model is exploratory throughout. Multiple comparisons were made without formal adjustment; borderline p-values should be treated with caution.

### DRC subnational analysis

Health zones were classified using two prespecified 75th percentile thresholds across all 200 zones: cumulative reported cases ≥992 and recent 12-month reported cases ≥36. Persistent hotspots had high cumulative and high recent burden (≥992 cumulative cases and ≥36 recent cases). Emerging hotspots had lower cumulative but high recent burden (<992 cumulative cases and ≥36 recent cases). Historical hotspots had high cumulative but lower recent burden (≥992 cumulative cases and <36 recent cases). Low-burden zones had lower cumulative and lower recent burden (<992 cumulative cases and <36 recent cases). Eastern provinces (Sud-Kivu, Nord Kivu, Maniema, Tanganyika, Haut Lomami, Haut Katanga, and Lualaba) were used as a geographic proxy for clade Ib distribution because zone-level clade confirmation data were not available. DRC subnational CFRs are based on total reported health-zone cases aggregating suspected/clinically compatible and confirmed cases; they are not comparable to confirmed-only CFRs.

Supplementary analyses. Event-study windows and the March 2025 ecological comparison contrasted January 2024 to February 2025 with March 2025 to February 2026 as an operational descriptive boundary around the subsequent higher-burden period. The March 2025 ecological comparison was retained as an exploratory supplementary analysis because this period coincided with documented reductions and uncertainty in external health financing and response support [23–25]. This comparison was treated as hypothesis-generating only and was not interpreted as evidence of a financing effect on mpox trajectory. Results are presented in the supplementary materials. Analysis was conducted using Python 3.12; pandas 3.0.2, numpy 2.4.4, statsmodels 0.14.6, scipy 1.17.1, and networkx 3.6.1.

### Ethical statement

This study used publicly available aggregate surveillance data. No individual-level data were accessed and formal ethics review was not required.

## Results

### Global epidemic trajectory and phase-specific burden

Between January 2024 and February 2026, 82,738 confirmed mpox cases and 306 deaths (overall phase-level case fatality ratio (CFR) 0.37%) were reported to WHO globally (Table 1; Fig 1). Countries reporting cases numbered 31–67 per month, with the highest count in August 2025 (n = 67). Monthly CFR estimates are volatile because of the small number of deaths relative to cases in individual months and should be interpreted at the phase level rather than month by month.

**Table 1:** Phase-specific global mpox burden, January 2024 to February 2026.

Phase	Months	Cases (n)	Deaths (n)	CFR (%)	Peak month (cases)	Median monthly cases (IQR)	Countries reporting (median)
Pre-PHEIC (Jan-Aug 2024)	8	11,403	48	0.42	Aug 2024 (2,311)	1,203 (1,026−1,662)	38
PHEIC (Sep 2024-Sep 2025)	13	61,220	223	0.36	May 2025 (7,405)	4,626 (3,943−4,859)	52
Post-PHEIC (Oct 2025-Feb 2026)	5	10,115	35	0.35	Oct 2025 (3,201)	1,610 (1,524−2,596)	49
**Total (Jan 2024-Feb 2026)**	**26**	**82,738**	**306**	**0.37**	**May 2025 (7,405)**	–	–

Global confirmed case and death counts by phase (confirmed cases only). CFR = phase-level deaths/cases; monthly CFR estimates are volatile given small death counts. PHEIC, Public Health Emergency of International Concern; PHECS, Africa CDC Public Health Emergency of Continental Security; IQR, interquartile range.

**Fig 1:**
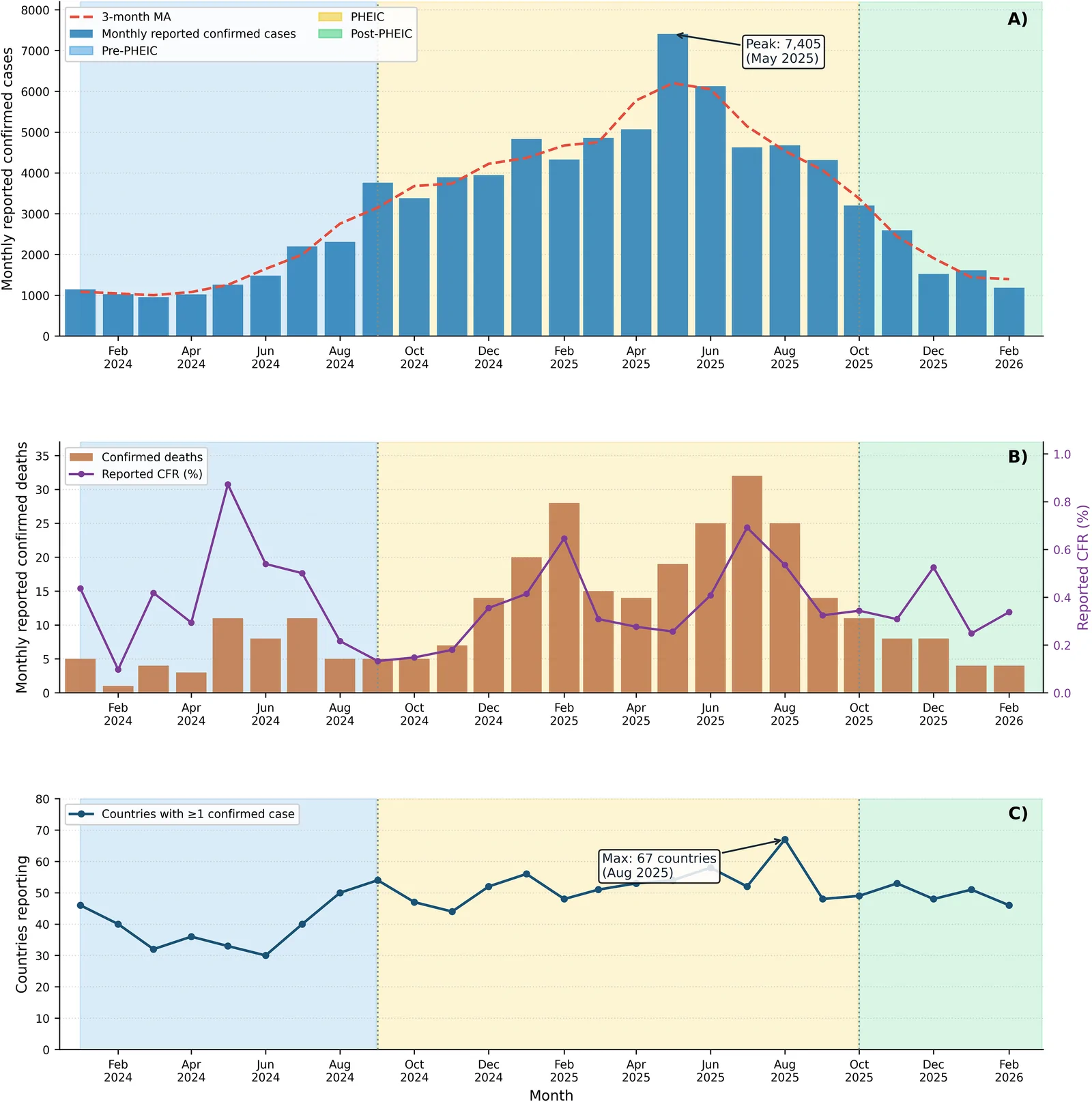
Global reported confirmed mpox cases, deaths, and reporting countries, January 2024 to February 2026. Panel A shows monthly reported confirmed cases and a 3-month moving average. Panel B shows monthly reported confirmed deaths and monthly reported CFR, calculated as deaths divided by confirmed cases reported in the same month. Panel C shows countries reporting at least one confirmed case per month. Phase shading indicates Pre-PHEIC, PHEIC, and Post-PHEIC periods; dotted vertical lines mark phase boundaries. CFR, case fatality ratio; PHEIC, WHO Public Health Emergency of International Concern.

The pre-PHEIC period (January to August 2024) accounted for 11,403 confirmed cases and 48 deaths (phase CFR 0.42%), with a median of 1,203 monthly cases (interquartile range (IQR) 1,026–1,662). The epidemic peak occurred in May 2025 with 7,405 confirmed cases during the PHEIC period, which accounted for 74.0% of all confirmed cases (61,220 cases; 223 deaths; phase CFR 0.36%). The post-PHEIC period accounted for 10,115 confirmed cases and 35 deaths (phase CFR 0.35%), with the Africa CDC PHECS subphase contributing 8,931 cases and the post-PHECS subphase 1,184 cases. Phase-level CFRs are descriptive reported surveillance ratios and should not be interpreted as true lethality estimates.

A two-breakpoint NB interrupted time-series (ITS) model identified an immediate upward shift in the global reported case rate at the PHEIC declaration breakpoint (reported case-rate ratio (CRR) 1.499, 95% CI 1.095–2.052; p = 0.011), followed by a non-significant trend toward monthly deceleration during the PHEIC period (slope CRR 0.933 per month, 95% CI 0.866–1.005; p = 0.066). This upward shift reflects continued escalation of reported transmission already underway at the time of declaration. An additional upward shift was identified at the April 2025 SPRP breakpoint (CRR 1.607, 95% CI 1.168–2.211; p = 0.004), followed by a steep monthly decline (slope CRR 0.796 per month, 95% CI 0.745–0.851; p < 0.001) (Table 2). Because the April 2025 breakpoint falls one month before the May 2025 global case peak, the post-breakpoint decline largely reflects the natural post-peak descent of the epidemic curve rather than any SPRP effect. In sensitivity analyses, the April 2025 breakpoint was shifted by minus one month (March 2025), plus one month (May 2025), and plus two months (June 2025); the post-breakpoint slope remained consistently below 0.82 and highly significant (p < 0.001).

**Table 2:** Segmented negative binomial regression results for reported mpox confirmed case rates around prespecified policy-response breakpoints, January 2024 to February 2026.

Model / Milestone	Parameter	Estimate (CRR)	95% CI lower	95% CI upper	p-value
***SEGMENTED REGRESSION — Global monthly confirmed cases***					
PHEIC declaration level shift	Level CRR	1.499	1.095	2.052	0.011
Within-PHEIC monthly slope	Slope CRR/month	0.933	0.866	1.005	0.066
April 2025 SPRP level shift	Level CRR	1.607	1.168	2.211	0.004
Post-April 2025 monthly slope	Slope CRR/month	0.796	0.745	0.851	<0.001
***SEGMENTED REGRESSION — Africa weekly confirmed cases***					
PHEIC declaration level shift	Level CRR	2.673	1.612	4.432	<0.001
Within-PHEIC weekly slope	Slope CRR/week	1.042	1.016	1.069	0.002
April 2025 SPRP level shift	Level CRR	1.469	0.879	2.453	0.142
Post-April 2025 weekly slope	Slope CRR/week	0.924	0.902	0.945	<0.001
***BREAKPOINT SENSITIVITY — Global monthly model, post-breakpoint slope***					
March 2025 (−1 month)	Slope CRR/month	0.817	0.742	0.899	<0.001
April 2025 (primary)	Slope CRR/month	0.796	0.745	0.851	<0.001
May 2025 (+1 month)	Slope CRR/month	0.776	0.741	0.812	<0.001
June 2025 (+2 months)	Slope CRR/month	0.753	0.718	0.790	<0.001

(i) NB, negative binomial; ITS, interrupted time-series; PHEIC, Public Health Emergency of International Concern; SPRP, Strategic Preparedness and Response Plan; CRR, reported case-rate ratio from the NB model with log-population offset. Values above 1.0 indicate a higher reported case rate than the prior trend; values below 1.0 indicate a lower reported case rate. (ii) Level CRR: immediate step change in reported case rate at the breakpoint, after accounting for the pre-existing trend. (iii) Slope CRR per month or week: incremental change in reported case rate per time unit after the breakpoint. The April 2025 breakpoint falls one month before the May 2025 global peak; the post-April slope largely reflects the natural post-peak epidemic decline. (iv) Eight-week event-study windows around selected policy milestones are presented in S1 Table as descriptive trajectory summaries without inferential emphasis.

### Regional distribution

The WHO African Region (AFRO) dominated the globally reported confirmed case burden throughout the study period. Its share rose from 20.0% in January 2024 to 88.3% at the May 2025 peak, remaining at 58.6% in February 2026. AFRO contributed 49,101 confirmed cases during the PHEIC period, representing a mean monthly share of 79.0%. Confirmed case trends across WHO regions are shown in S1 Fig.

### African weekly surveillance dynamics

Weekly reported confirmed mpox cases from the WHO African surveillance series (112 weeks within the primary analytic cutoff) showed that the PHEIC declaration was associated with an immediate increase in the reported weekly confirmed case rate (CRR 2.673, 95% CI 1.612–4.432; p < 0.001; Fig 2). A progressive weekly increase in the reported case rate continued during the PHEIC period (slope CRR 1.042 per week, 95% CI 1.016–1.069; p = 0.002), while the level shift at the April 2025 SPRP breakpoint was not statistically significant in the African weekly model (CRR 1.469, 95% CI 0.879–2.453; p = 0.142). A significant decline in reported weekly confirmed cases was observed after the April 2025 breakpoint (slope CRR 0.924 per week, 95% CI 0.902–0.945; p < 0.001), which largely reflects the post-peak descent of the epidemic curve rather than an effect of the SPRP update (Table 2).

**Fig 2:**
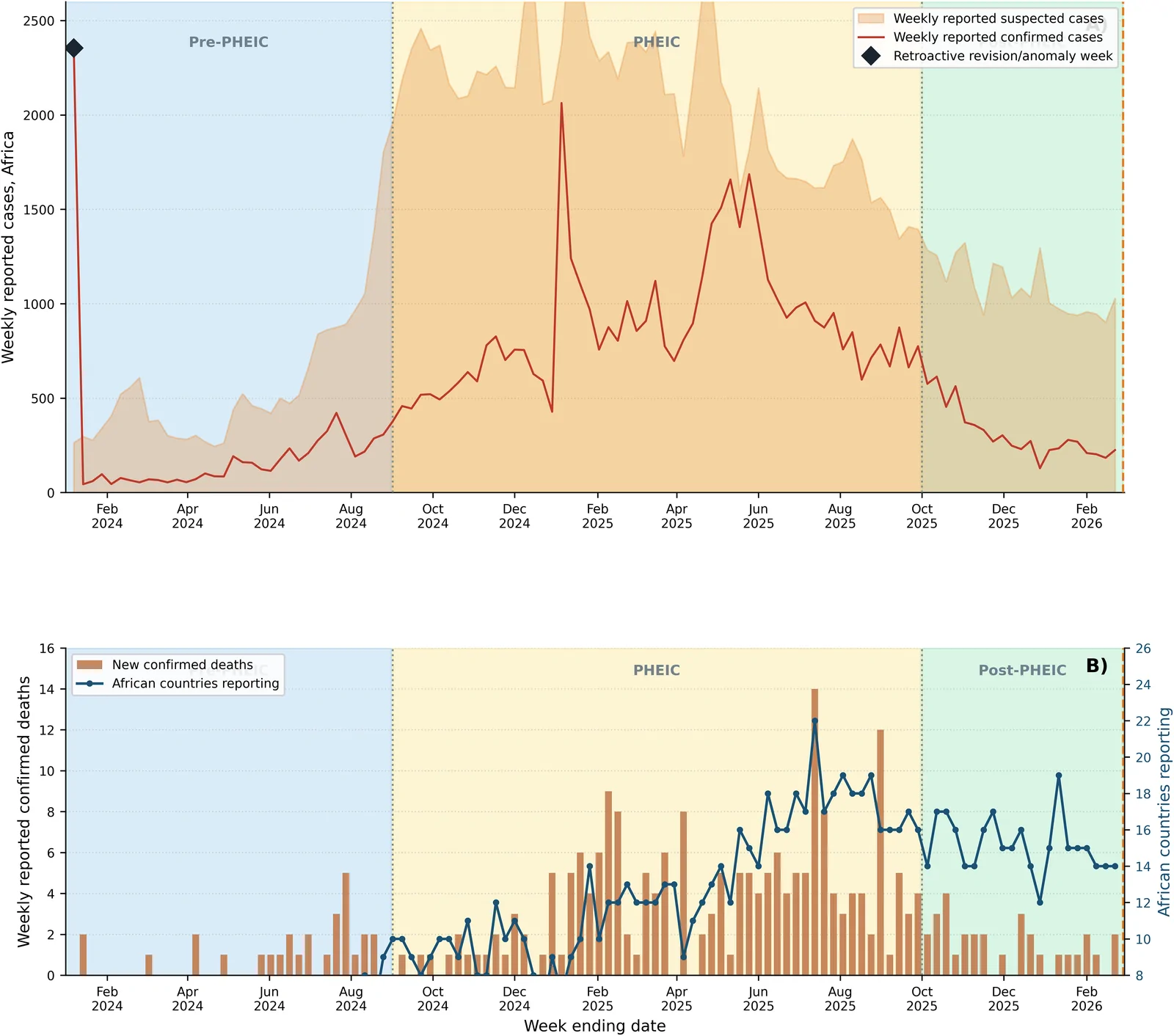
African weekly mpox surveillance trends, January 2024 to February 2026. Panel A shows weekly reported confirmed and suspected cases in Africa. Diamond markers indicate validated retroactive revision/anomaly weeks. Panel B shows weekly reported confirmed deaths (bars, left axis) and the number of African countries reporting cases in the weekly surveillance series (line, right axis). The orange dashed line marks the 28 February 2026 primary analytic cutoff. Confirmed deaths reflect deaths among confirmed cases only; suspected-case deaths are not included. PHEIC, WHO Public Health Emergency of International Concern.

### International clade importation and documented secondary transmission

A total of 163 clade-specific importation or travel-linked detection events were documented within the primary analytic cutoff. These records represent documented detections rather than all international importations, as detection depends on testing capacity, clade confirmation, and public reporting in each destination country. Clade Ib accounted for 155 events (95.1%), clade Ia for four (2.5%), and Other/Uncertain for four (2.5%), including two recombinant Ib/IIb events. Events were documented across 41 countries. EURO recorded the most events (73, 44.8%), followed by SEARO (34, 20.9%), AFRO (22, 13.5%), WPRO (15, 9.2%), AMRO (11, 6.7%), and EMRO (8, 4.9%).

Of 163 documented records, 25 (15.3%) had documented secondary transmission (ST), defined as any documented onward case or cases following a travel-linked detection event; 24 of 25 ST events were clade Ib and one was clade I without subclade specification, occurring across 16 of 41 countries (Table 3). ST captures any event with at least one onward case and does not distinguish isolated secondary cases from sustained local spread; it is a documented surveillance outcome rather than a measure of transmissibility. The ST rate was higher during the PHEIC phase than in the post-PHEIC period (18/93 events, 19.4% vs 7/70 events, 10.0%). WPRO had the highest ST rate (7/15, 46.7%), followed by EMRO (3/8, 37.5%), AFRO (6/22, 27.3%), EURO (8/73, 11.0%), SEARO (1/34, 2.9%), and AMRO (0/11, 0.0%). In an exploratory logistic regression of binary ST status against destination region and origin group, with EURO as the reference region for destination and non-Africa-origin as the reference group for origin, AMRO was not estimable because no ST events occurred among AMRO-destination records (complete separation). Among estimable destination-region comparisons, WPRO showed the strongest association with documented ST (OR 8.780, 95% CI 2.295–33.582; p = 0.002). EMRO showed a borderline association (OR 4.624, 95% CI 0.920–23.246; p = 0.063). AFRO did not meet the conventional significance threshold (OR 2.774, 95% CI 0.795–9.680; p = 0.110). SEARO showed lower odds than EURO (OR 0.285, 95% CI 0.032–2.546; p = 0.261). Africa-origin events showed a non-significant trend toward higher ST rates compared with non-Africa-origin events in a supplementary Fisher exact test (OR 2.515; p = 0.051; Africa-origin 17/77 events [22.1%] vs non-Africa-origin 8/79 events [10.1%]). All ST association findings are exploratory, subject to instability from small event counts, and should be interpreted with caution; multiple comparisons were made without formal adjustment (Table 3).

**Table 3:** International mpox travel-linked detection events and documented secondary transmission, by destination WHO region and origin group, August 2024 to February 2026.

Category	Events (n)	Clade Ib (%)	ST events (n)	ST rate (%)	OR vs EURO or non-Africa (95% CI; p)
**DESTINATION REGION**					
AFRO	22	100	6	27.3	2.774 (0.795–9.680); p = 0.110
AMRO	11	100	0	0.0	Complete separation
EMRO	8	100	3	37.5	4.624 (0.920–23.246); p = 0.063
EURO (reference)	73	95	8	11.0	1.00 (ref)
SEARO	34	94	1	2.9	0.285 (0.032–2.546); p = 0.261
WPRO	15	87	7	46.7	8.780 (2.295–33.582); p = 0.002
**Total**	**163**	**95**	**25**	**15.3**	–
**ORIGIN GROUP**					
Africa-origin (all)	77	100	17	22.1	OR 2.515; p = 0.051 (Fisher exact test)
Non-Africa origin	79	95	8	10.1	Reference for Africa vs non-Africa comparison
Under investigation	7	–	0	0.0	Excluded from comparisons

ST, documented secondary transmission, defined as any documented onward case or cases following a travel-linked detection event; it does not distinguish isolated secondary cases from sustained local spread. AFRO, WHO African Region; AMRO, WHO Region of the Americas; EMRO, WHO Eastern Mediterranean Region; EURO, WHO European Region; SEARO, WHO South-East Asia Region; WPRO, WHO Western Pacific Region; OR, odds ratio. Destination-region ORs are from exploratory logistic regression with EURO as the reference region; AMRO was not estimable because no ST events were observed among AMRO-destination records. The Africa versus non-Africa origin OR is from Fisher exact testing and is presented as a supplementary descriptive comparison. All findings are exploratory; multiple comparisons were made without formal adjustment; borderline p-values should be interpreted with caution. Clade Ib percentage uses all events in the category as the denominator. Non-Ib records include clade Ia, clade I without subclade specification, and recombinant Ib/IIb records. Origin group was classified from reported place of origin, which reflects reported travel history and does not confirm the site of infection or transmission.

The United Arab Emirates (UAE) was the most frequent reported place of origin, appearing in 33 events linking to 11 destination countries. Place of origin reflects reported travel history and does not confirm the site of infection or transmission; the prominence of the UAE as a reported transit point may partly reflect its role as a major international aviation hub rather than local epidemiological amplification, and transit-country effects cannot be distinguished from source-country exposure without detailed contact investigation or genomic data. Documented direct DRC-origin links declined progressively, from 50% of events in Q3 2024 to zero by Q4 2025, while early documented events were more often linked to East African travel histories than to direct DRC origin (S2 Fig).

### Contextual global demographic profile: Aggregated surveillance data, 2022 to March 2026

Age and sex data were available for 99,463 confirmed cases; all figures below refer to confirmed cases unless otherwise stated. Males accounted for 96,199 cases (96.7%), corresponding to a male-to-female ratio of approximately 29:1. Males aged 20–49 years represented 85,876 cases (86.3%), and males aged 30–39 years were the largest single age-sex category (40,112 cases, 40.3%). This global profile is strongly shaped by the 2022–2023 clade IIb outbreak among adult men in non-African settings [3] and should not be generalised to African clade I or clade Ib outbreaks, where women and children account for a larger share of reported cases and where household, zoonotic, and community transmission occur alongside sexual transmission [8,26].

The recent 12-month period (ending March 2026) showed a higher proportion of cases with reported MSM status (92.2%), reported sexual transmission (96.0%), and reported travel history (25.7%) compared with the earlier cumulative period (87.3%, 89.0%, and 17.3%, respectively). Reported PLHIV status was proportionally lower in the recent period (40.7% vs 50.8% cumulatively). Hospitalisation rates were similar across periods (approximately 9%), and ICU admission remained rare. No deaths were recorded in the recent-period case subset, compared with 201 deaths in the earlier cumulative subset. These observed shifts in proportions should be interpreted with considerable caution. The recent-period case subset represents a small and non-random fraction of the cumulative total, with different reporting completeness, surveillance coverage, and geographic mix.

### Policy-response timeline and interrupted time-series findings

Thirty policy and response events were documented within the primary analytic cutoff (S3 Fig). These included WHO PHEIC declaration and termination, Africa CDC PHECS lifting, IHR Emergency Committee milestones, SPRP updates, rapid risk assessments, vaccine authorisations and emergency listings, technical vaccine guidance, and major vaccine allocation, rollout, and delivery milestones. Selected policy milestones and corresponding interrupted time-series findings are presented in Table 2.

### DRC sub-national burden

Within DRC, 200 health zones across 25 provinces recorded 144,035 total reported mpox cases and 2,047 deaths between the start of the outbreak and March 2026. These figures represent aggregates of clinically compatible, suspected, and confirmed cases at health-zone level without providing a confirmed-case breakdown. The overall subnational case fatality ratio (CFR) was 1.42%. Eastern provinces (Sud-Kivu, Nord Kivu, Maniema, Tanganyika, Haut Lomami, Haut Katanga, and Lualaba; 54 zones) accounted for 50,574 cases (35.1%) and 66 deaths, yielding a zone-aggregated CFR of 0.13%, compared with 2.12% in non-eastern provinces (146 zones; 93,461 cases; 1,981 deaths). This eastern versus non-eastern CFR contrast should be interpreted as a surveillance completeness artefact rather than an epidemiological severity signal, because zone-level clade confirmation and confirmed/suspected disaggregation were unavailable. The overall burden was geographically concentrated: the top five health zones accounted for 19.3% of all cases (27,854 cases), the top ten for 29.8% (42,957 cases), and the top 20 for 45.4% (65,322 cases). Miti-Murrhesa health zone (Sud-Kivu) recorded the highest cumulative case count (9,695 cases; zone CFR 0.11%). Health zones were classified as persistent hotspot (n = 39), emerging hotspot (n = 12), historical hotspot (n = 12), or low-burden (n = 137) using cumulative and recent 12-month case-count percentiles (75th percentile thresholds). This classification identified a core of 39 persistently active zones: 13 in eastern provinces and 26 in non-eastern provinces, alongside 12 zones with emerging recent burden and 12 with historical but currently lower activity. All 200 zones recorded at least one case in the most recent 12-month window, reflecting the geographically widespread nature of reported burden across DRC (S2 Table; Fig 3).

**Fig 3:**
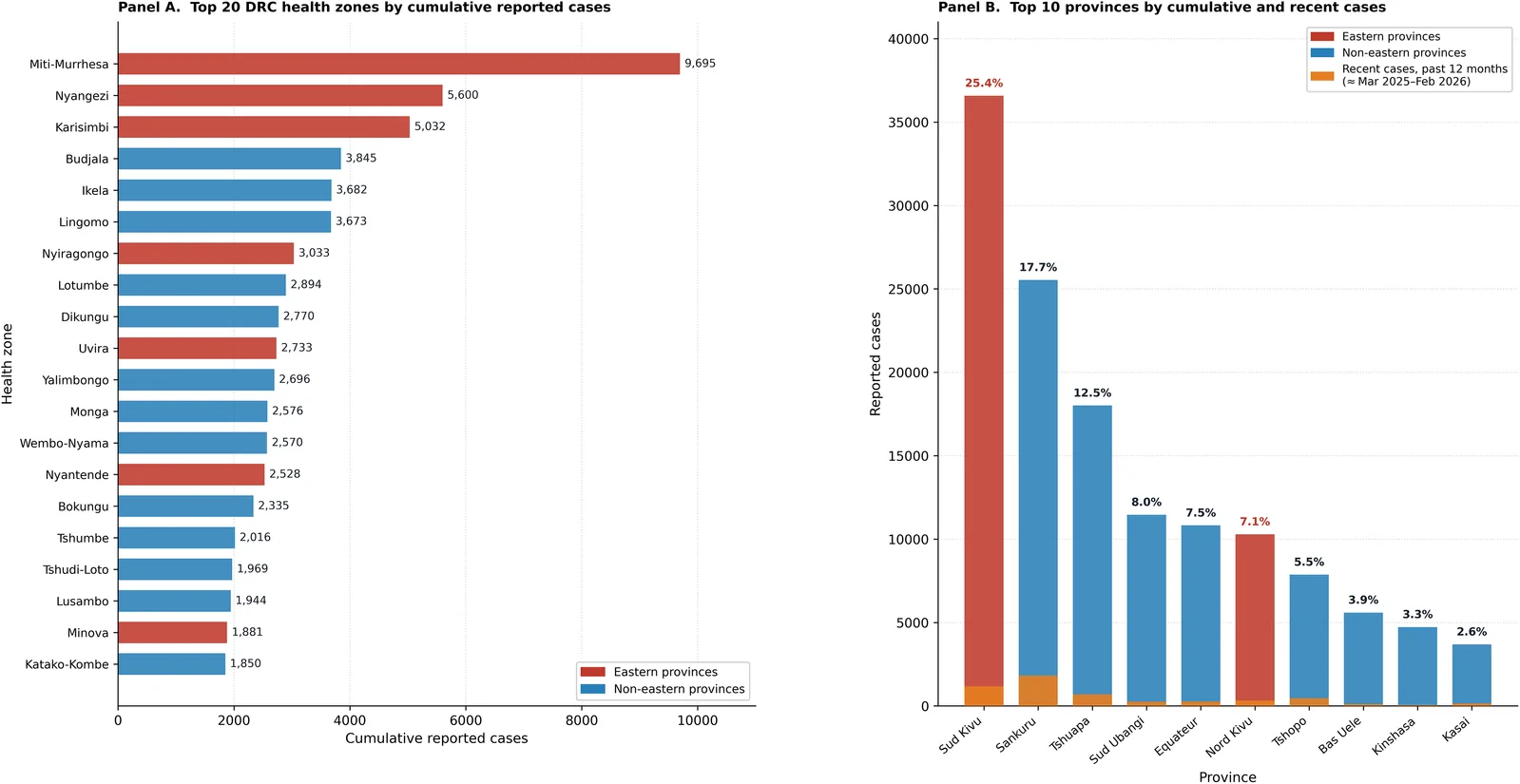
DRC health-zone and province-level mpox burden, January 2024 to March 2026. Panel A shows the top 20 DRC health zones by cumulative reported cases. Panel B shows the top 10 DRC provinces by cumulative and recent 12-month reported cases. Red indicates health zones or provinces within the seven-province eastern DRC proxy; blue indicates non-eastern provinces; orange bars show recent 12-month cases. Percentage labels indicate each province’s share of total cumulative DRC reported cases. Data represent aggregate health-zone surveillance reports comprising clinically compatible, suspected, and confirmed cases; no confirmed/suspected breakdown is publicly available at this level. DRC, Democratic Republic of the Congo.

### Exploratory comparison around the March 2025 analytic boundary

An exploratory ecological comparison around March 2025 is presented as a supplementary analysis (S4 Fig; S3 Table). It compares reported confirmed case burden before and after the start of the higher-burden period leading toward the May 2025 peak; findings are hypothesis-generating and should not be interpreted as causal effects of financing, policy, or response changes.

## Discussion

This surveillance analysis characterises the second mpox PHEIC cycle from the emergence of clade Ib in early 2024 through the post-emergency transition in February 2026. While recent syntheses have described the African epidemiology of the second PHEIC [10,11], the present analysis adds an integrated multi-domain framework spanning global and Africa-specific time-series modelling, DRC subnational burden, international travel-linked detection pathways, and the immediate post-emergency transition within a single structured analysis, including a 26-month monthly global series with segmented regression and a 112-week African weekly series with sensitivity analysis of the April 2025 breakpoint. Three findings stand out.

### Epidemic trajectory and temporal associations

The epidemic followed an escalation-peak-deceleration pattern across the study period. The PHEIC declaration coincided with an immediate rise in reported confirmed cases, reflecting continued transmission escalation alongside intensification of surveillance, case-finding, and international reporting associated with the emergency [27]; the PHEIC declaration should not be understood as the cause of this increase. The global model identified an upward level shift at the April 2025 SPRP breakpoint followed by the steepest monthly decline in the model series. However, this April 2025 breakpoint falls one month before the May 2025 global epidemic peak: the decline identified in the model therefore largely reflects the natural post-peak descent of the epidemic curve rather than an effect of the SPRP update. By the time of PHEIC termination, reported case rates were already declining, so termination should be interpreted as an administrative transition rather than an epidemiological endpoint. These temporal associations support interpreting the PHEIC as a governance and coordination instrument rather than a direct epidemiological intervention, and underline that emergency declaration and termination timings are driven by governance considerations that do not necessarily coincide with epidemic inflection points.

All model estimates are based on reported confirmed case counts from a heterogeneous global surveillance system; therefore, changes in reported case rates may reflect both transmission dynamics and changes in testing, ascertainment, and reporting.

### African burden, DRC subnational patterns, and clade biology

Reported confirmed cases became strongly concentrated in Africa across the study period, rising to 88.3% at the May 2025 peak and remaining at 58.6% in February 2026, contrasting with the predominantly non-African distribution of the 2022–2023 clade IIb outbreak [3]. This burden was multi-clade: clade IIb persisted in non-African and West African settings [10,11], clade Ia remained important in historically affected Central African areas [11], and clade Ib spread regionally and internationally from eastern DRC [8,9]. Although all presentations are classified as mpox, clade attribution remains important for surveillance, severity interpretation, and response planning.

The substantially lower zone-aggregated CFR in eastern DRC provinces (0.13%) compared with non-eastern provinces (2.12%) has previously been discussed in relation to possible clade Ib characteristics [26]. In this analysis, it is more conservatively interpreted as a surveillance-completeness artefact rather than an epidemiological severity signal. Greater ascertainment of suspected and mild cases in intensively monitored eastern provinces would mechanically lower the calculated CFR, and age-adjusted, severity-stratified, and confirmed-only provincial data were not available.

More broadly, current evidence on clinical differences between mpox clades at the population level remains limited. Clade Ib outbreaks in eastern DRC have been characterised by broader age and sex distribution and predominantly non-sexual community and household transmission patterns compared with clade IIb, which has been concentrated in MSM networks with predominantly mild outcomes in immunocompetent adults in non-African settings [3,4,8]. Clade Ia has historically been reported in Central African settings with higher observed CFRs [6,8,11], particularly among children, although these estimates are also affected by surveillance intensity, case ascertainment, and care-access differences. Population-level CFR estimates from aggregate surveillance data should therefore not be used to draw conclusions about clade-specific virulence without individual-level clinical data.

### International spread and secondary transmission

Documented travel-linked detection events were identified across 41 countries, with secondary transmission documented in 25 of 163 events (15.3%). Destination region was the strongest predictor in the exploratory model, with WPRO showing the highest odds of documented secondary transmission relative to EURO. These regional differences most likely reflect a combination of testing intensity, contact-tracing capacity, surveillance completeness, and differences in contact structure, rather than a single biological mechanism. The higher ST signal among Africa-origin events should be interpreted cautiously. It may reflect a combination of underlying transmission context, exposure setting, ascertainment, and contact-tracing intensity rather than an intrinsic biological difference in Africa-originating events. This interpretation is consistent with recent descriptions of travel-related clade Ib detections, which emphasise documented transmission patterns but do not establish comparative transmissibility across origins [8,28].

The decline in directly DRC-linked events over time and the shift toward East African travel-associated and non-African-origin events suggest that post-emergency surveillance should not be focused exclusively on DRC-origin importations. Clinicians outside endemic regions should be aware that travel history to sub-Saharan Africa or East Africa – not only DRC – and MSM status are the predominant reported risk factors in non-African settings, while presentations in African settings follow fundamentally different demographic and transmission profiles characterised by broader age and sex distributions, household contacts, and non-sexual transmission routes.

### Surveillance bias as a central interpretive constraint

A major interpretive constraint of this analysis is that all findings are based on aggregate reported surveillance data from a heterogeneous global system with variable testing capacity, differing case definitions, and changing reporting practices over time and across countries. The increases in reported case counts following the PHEIC declaration may partly reflect expanded testing, broader case definitions, and increased clinician awareness rather than true increases in incidence. Conversely, subsequent declines may reflect surveillance fatigue, reporting normalisation, or reductions in testing activity rather than solely true epidemic decline. Testing volumes and positivity rates were not available consistently in the public datasets and could not be used to adjust these estimates. These surveillance limitations affect the interpretation of all reported case-rate comparisons, regional differences, and temporal associations described in this analysis, and are treated as central rather than peripheral interpretive constraints throughout.

### Financing context

March 2025 was used only as an operational descriptive boundary in S3 Table and S4 Fig. It also coincided with documented reductions and uncertainty in external health financing and response support affecting some settings [23–25]. Because the post-March period overlaps with the approach to the May 2025 peak, any contribution of financing disruptions remains unproven and hypothesis-generating.

### Strengths and limitations

Strengths. This analysis integrates global monthly and African weekly surveillance time-series, DRC subnational health-zone burden, international travel-linked detection pathways, and demographic context across the full second PHEIC cycle and immediate post-emergency transition within a single structured analysis. The segmented negative binomial regression framework with log-population offsets is appropriate for overdispersed count data, and the two policy-driven breakpoints were selected on substantive grounds rather than by post-hoc data mining. Sensitivity analysis shifting the April 2025 breakpoint by one and two months confirmed consistent direction and significance of post-peak slope estimates.

Limitations. Several limitations affect interpretation. First and most fundamentally, all analyses rely on aggregate surveillance data with heterogeneous reporting practices, variable testing capacity, and differing case definitions across countries and time periods. Changes in reported case rates over time reflect both transmission dynamics and surveillance intensity, and these cannot be disentangled from aggregate data alone. Testing volumes and positivity rates were not available consistently in the public datasets, preventing adjustment for differential ascertainment. Countries undergoing major surveillance system changes during the study period cannot be identified from the available aggregate data. Second, demographic and epidemiological characteristic data were available only as cumulative global figures from 2022 to March 2026 and could not be restricted to the primary study period. Third, the importation and secondary transmission analysis is based on 163 WHO-tracked events and 25 secondary transmission outcomes; the exploratory logistic regression has a low events-per-variable ratio and wide confidence intervals, and AMRO showed complete separation. Fourth, DRC subnational CFRs are calculated from total reported health-zone cases aggregating suspected and confirmed cases without a confirmed-case breakdown, and should not be interpreted as true lethality estimates. Fifth, zone-level clade confirmation data were not available, preventing direct attribution of subnational patterns to specific clades. Sixth, age-adjusted or severity-stratified data were not available at the regional or provincial level, limiting interpretation of CFR differences across settings. Seventh, the interrupted time-series models identify temporal associations around prespecified policy-response breakpoints and cannot establish causal effects; in particular, the April 2025 breakpoint falls one month before the May 2025 epidemic peak, so the steep post-April decline largely reflects the natural post-peak descent rather than any SPRP effect.

## Conclusion

Emergency termination should be understood as a shift from acute global alert to sustained response, not as epidemic resolution. The second PHEIC cycle showed that clade Ib was not confined to its original epicentre: it spread internationally, concentrated reported burden in Africa and persistent DRC hotspot zones, and sustained transmission beyond DRC. The disproportionate burden in Africa and the continuing risk of international re-seeding require coordinated action beyond the PHEIC framework. The next phase requires resilient surveillance, sustained diagnostic capacity, community-engaged risk communication, targeted action in persistent DRC hotspots and affected regional corridors, and equitable access to vaccines and clinical care.

## Use of artificial intelligence tools

The author used Claude (Anthropic) during preparation of this work for language refinement, structural editing, internal consistency checking, and code review support. The author reviewed and edited all AI-assisted outputs, verified all factual claims, references, and numerical results against primary sources, and takes full responsibility for the content, analysis, interpretation, and conclusions. AI was not used to generate original data, perform independent analyses, or draw scientific conclusions.

## Supporting information

S1 Checklist STROBE checklist for observational surveillance study reporting. (PDF)

S1 Table Descriptive pre-/post-window changes in reported confirmed mpox cases around selected policy milestones. (DOCX)

S2 Table Top 20 DRC health zones by cumulative reported mpox burden and hotspot typology, onset of outbreak to mid-March 2026. (DOCX)

S3 Table Exploratory ecological comparison of reported mpox indicators before and after the March 2025 analytic boundary. (DOCX)

S4 Table Demographic and epidemiological characteristics, cumulative and recent period comparisons, 2022-March 2026. (DOCX)

S1 Fig Monthly reported confirmed mpox cases by WHO region, January 2024 to February 2026. Panels A–F show monthly reported confirmed mpox cases (bars, left axis) and reported confirmed cases per 1 million population (dashed line, right axis) for each of the six WHO regions. Phase shading reflects epidemic phase boundaries used in the monthly analysis: blue, Pre-PHEIC (January to August 2024); yellow, PHEIC (September 2024 to September 2025); green, Post-PHEIC (October 2025 to February 2026). Y-axis scales differ across panels to allow within-region trends to be visible; AFRO operates at a substantially larger absolute scale than the other regions. Confirmed case counts are from the WHO Global Mpox Trends dashboard, downloaded in April 2026, with a primary analytic cutoff of 28 February 2026. Population denominators are from the United Nations Population Division World Population Prospects 2024.
(TIFF)

S2 Fig Quarterly distribution and share of DRC-origin and Africa-origin travel-linked detection events, 2024–2026. Panel A shows quarterly importation or travel-linked detection events stacked by reported place of origin: DRC, Africa non-DRC, non-Africa, and under investigation. Percentages above bars indicate the DRC-origin share of events with known origin, excluding under-investigation events from the denominator. Panel B shows the DRC-origin share and Africa-origin share of events with known origin over time (left axis), alongside documented secondary transmission (ST) events per quarter (bars, right axis). ST was defined as any documented onward case or cases following a travel-linked detection event. Seven events with origin under investigation were excluded from origin-share calculations. The analysis was restricted to 163 clade-specific travel-linked detection records within the primary analytic cutoff of 28 February 2026. Place of origin reflects reported travel history and does not confirm the site of infection or transmission. Quarter definitions: Q1, January–March; Q2, April–June; Q3, July–September; Q4, October–December.
(TIFF)

S3 Fig Integrated epidemic and policy-response timeline, January 2024 to February 2026. Panel A shows monthly global reported confirmed mpox cases (bars) with a 3-month moving average (dashed line) and coloured vertical markers indicating documented policy and response events captured in the analytic database, colour-coded by category. Panel B shows weekly reported confirmed mpox cases in Africa (red line), with weekly reported suspected cases shown as orange background shading for surveillance context only; suspected cases were not included in the segmented regression models. Five priority policy milestones are annotated. Panel C shows the policy-response event timeline containing all 30 documented events captured in the analytic database within the primary analytic cutoff of 28 February 2026, plotted by category using distinct marker shapes and colours. Phase shading across all panels indicates the epidemic phases used in the analysis: blue, Pre-PHEIC (January to August 2024); yellow, PHEIC (September 2024 to September 2025); green, Post-PHEIC (October 2025 to February 2026). Overlapping milestone timing precludes attribution of trajectory changes to any single event. PHEIC, WHO Public Health Emergency of International Concern; PHECS, Africa CDC Public Health Emergency of Continental Security; IHR, International Health Regulations (2005); SPRP, WHO Strategic Preparedness and Response Plan; MVA-BN, Modified Vaccinia Ankara-Bavarian Nordic vaccine.
(TIFF)

S4 Fig Descriptive comparison of reported confirmed mpox case burden before and after March 2025, January 2024 to February 2026. Panel A shows monthly global reported confirmed mpox cases with pre- and post-boundary medians. Panel B shows weekly reported confirmed mpox cases in the WHO African Region, with weekly reported suspected cases shown as contextual shading only; suspected cases were not included in confirmed-case regression models. Panel C shows monthly global reported confirmed cases stacked by WHO region, with labels indicating AFRO’s share in selected months. The dark dashed vertical line marks March 2025, used as an operational boundary separating January 2024-February 2025 from March 2025-February 2026 for descriptive comparison of reported confirmed case burden. March 2025 is not a policy event, financing marker, intervention date, or causal breakpoint. Percentage annotations show descriptive median differences between periods. Phase shading: blue, Pre-PHEIC (January to August 2024); yellow, PHEIC (September 2024 to September 2025); green, Post-PHEIC (October 2025 to February 2026). AFRO, WHO African Region; AMRO, WHO Region of the Americas; EURO, WHO European Region; WPRO, WHO Western Pacific Region; SEARO, WHO South-East Asia Region; EMRO, WHO Eastern Mediterranean Region; PHEIC, WHO Public Health Emergency of International Concern.
(TIFF)
