# Supplementary material for: Global mpox outbreak dynamics during and after the 2024–2026 public health emergency
Source: PLoS One. 2026 Oct 21;21(10):e0350572. doi: 10.1371/journal.pone.0350572 (PMC13686470; doi:10.1371/journal.pone.0350572)
Supplement: Supplementary file 1 [file pone.0350572.s001.pdf]

S1 Checklist. STROBE checklist for observational surveillance study reporting

Strengthening the Reporting of Observational Studies in Epidemiology (STROBE) Statement: checklist of items included in this retrospective surveillance analysis. Items indicate where each reporting element is addressed in the revised manuscript.

Section	Item	Recommendation	Where addressed in manuscript
TITLE AND ABSTRACT	1a	Indicate the study design in the title or abstract.	Title states the topic and period; the abstract identifies the work as a retrospective surveillance analysis.
TITLE AND ABSTRACT	1b	Provide an informative and balanced summary of what was done and what was found.	Abstract summarises the study period, data sources, analytic approaches, principal numerical findings, and interpretation of the post-emergency transition.
INTRODUCTION	2	Explain the scientific background and rationale.	Introduction describes mpox background, clade Ib emergence, the 2022-2023 clade IIb outbreak context, prior PHEIC, and rationale for analysing the 2024-2026 emergency cycle.
INTRODUCTION	3	State specific objectives, including any pre-specified hypotheses.	Final Introduction paragraph states the objective: to integrate global and African surveillance trends, DRC subnational burden, and cross-border spread across the second emergency cycle and post-emergency transition.
METHODS	4	Present key elements of study design early in the paper.	Methods and abstract identify the study as a retrospective surveillance analysis using publicly available aggregate surveillance data.
METHODS	5	Describe the setting, locations, and relevant dates, including periods of recruitment, exposure, follow-up, and data collection.	Methods (Data sources and study period) describe the WHO Global Mpox Trends dashboard, WHO situation reports S32-S64, January 2024 to 28 February 2026 primary study period, and component-specific cutoffs through March 2026 where indicated.
METHODS	6a	Give eligibility criteria and the sources and methods of selection of participants.	Aggregate surveillance records were used. All available reported cases, deaths, health-zone records, and travel-linked detection events within the defined analytic windows and public data sources were included; no individual enrolment or exclusion was performed.
METHODS	7	Clearly define all outcomes, exposures, predictors, potential confounders, and effect modifiers.	Methods define confirmed case counts, deaths, CFR, reported case-rate ratios (CRRs), documented secondary transmission (ST), origin group, destination region, epidemic phase, DRC hotspot typology, eastern-province proxy, and March 2025 analytic boundary.

Section	Item	Recommendation	Where addressed in manuscript
METHODS	8	For each variable of interest, give sources of data and details of methods of assessment.	Methods identify the WHO Global Mpox Trends dashboard as the primary quantitative source and describe use of WHO situation reports, Africa CDC, US CDC, ECDC, national public health reports, and UN Population Division denominators for validation, context, and regression offsets.
METHODS	9	Describe any efforts to address potential sources of bias.	Methods and Discussion address potential bias through source validation, explicit case-definition separation, reported-rate terminology, and caution about retrospective revision, differential reporting completeness, testing capacity, surveillance intensity, and ecological interpretation.
METHODS	10	Explain how the study size was arrived at.	All available aggregate surveillance observations within the defined sources and analytic windows were included. No sample-size calculation was applicable for this retrospective surveillance analysis.
METHODS	11	Explain how quantitative variables were handled in the analyses. If applicable, describe which groupings were chosen and why.	Methods describe handling of quantitative variables using negative binomial models with log-population offsets, phase definitions, binary ST coding, destination and origin categories, Mann-Whitney U tests for exploratory ecological comparisons, and percentile-based DRC hotspot thresholds.
METHODS	12a	Describe all statistical methods, including those used to control for confounding.	Methods describe negative binomial segmented regression, overdispersion diagnostics, residual autocorrelation assessment, breakpoint sensitivity analyses, exploratory logistic regression for ST, directed travel-pathway network analysis, descriptive event windows, and Mann-Whitney U tests.
METHODS	12b	Describe any methods used to examine subgroups and interactions.	Methods and Results describe phase-based comparisons, destination-region and origin-group comparisons for ST, WHO regional distribution, eastern versus non-eastern DRC province contrasts, and DRC hotspot typology categories.
METHODS	12c	Explain how missing data were addressed.	No imputation was performed. Demographic and epidemiological variables were analysed among cases with available data for each variable, with variable-specific denominators reported in S4 Table and described in the contextual demographic Results section.
METHODS	12d	If applicable, explain how loss to follow-up was addressed.	Not applicable. This was an aggregate retrospective surveillance analysis without individual follow-up.

Section	Item	Recommendation	Where addressed in manuscript
METHODS	12e	Describe any sensitivity analyses.	Methods and Results describe sensitivity analyses shifting the April 2025 breakpoint by minus one, plus one, and plus two months. Exploratory descriptive event windows and March 2025 ecological comparisons are provided in supplementary tables and figures.
RESULTS	13a	Report numbers of individuals at each stage of the study.	Results report global totals (82,738 confirmed cases and 306 deaths), phase-specific burden, regional distribution, 163 travel-linked detection records, 25 ST events, 200 DRC health zones, and DRC health-zone/province burden.
RESULTS	13b	Give reasons for non-participation at each stage.	Not applicable. The study used aggregated surveillance data rather than participant recruitment.
RESULTS	14a	Give characteristics of study participants and information on exposures and potential confounders.	Results report phase-specific global burden (Table 1), contextual demographic and epidemiological characteristics, travel-linked detection characteristics (Table 3), and DRC subnational burden (Fig 3 and S2 Table).
RESULTS	14b	Indicate number of participants with missing data for each variable of interest.	Contextual demographic Results and S4 Table report variable-specific denominators for cases with available data and state that demographic data are cumulative from 2022 to March 2026 rather than restricted to the primary study period.
RESULTS	15	Report numbers of outcome events or summary measures.	Results present outcome counts and summary measures in Tables 1-3, Figs 1-3, S1-S4 Tables, and S1-S4 Figs.
RESULTS	16a	Give unadjusted estimates and, if applicable, confounder-adjusted estimates and their precision.	Results present CRRs with 95% CIs and p-values in Table 2, ORs with 95% CIs and p-values or Fisher exact testing in Table 3, and descriptive medians and p-values in S3 Table.
RESULTS	16b	Report category boundaries when continuous variables were categorised.	Methods and S2 Table note report DRC hotspot category boundaries: cumulative reported cases ≥992 and recent 12-month reported cases ≥36, with categories defined as persistent, emerging, historical, or low-burden.
RESULTS	16c	If relevant, consider translating estimates of relative risk into absolute risk for a meaningful time period.	Absolute case counts, deaths, CFRs, phase totals, travel-linked detection counts, ST counts, and DRC health-zone/province counts are reported alongside relative measures.
RESULTS	17	Report other analyses done, including subgroup analyses and sensitivity analyses.	Results report breakpoint sensitivity analyses (Table 2), descriptive event-study windows (S1 Table), ST logistic regression (Table 3), DRC hotspot classification (S2 Table), March 2025 ecological comparison (S3 Table and S4 Fig), and regional trend figures.

Section	Item	Recommendation	Where addressed in manuscript
DISCUSSION	18	Summarise key results with reference to study objectives.	Discussion summarises key findings on epidemic trajectory and emergency transition, Africa-centred multi-clade burden and DRC subnational patterns, international travel-linked detection with ST, surveillance bias, and financing context.
DISCUSSION	19	Discuss limitations, taking into account sources of potential bias or imprecision.	Strengths and limitations discuss aggregate surveillance data, heterogeneous testing and reporting, unavailable testing volumes and positivity rates, cumulative demographic denominators, limited ST events, DRC suspected/confirmed aggregation, unavailable zone-level clade confirmation, and non-causal ITS interpretation.
DISCUSSION	20	Give a cautious overall interpretation of results considering objectives, limitations, multiplicity of analyses, results from similar studies, and other relevant evidence.	Discussion interprets results cautiously, distinguishes reported case rates from true incidence, avoids causal interpretation of PHEIC/SPRP/March 2025 timing, treats ST as documented secondary transmission rather than sustained community spread, and frames exploratory analyses as hypothesis-generating.
DISCUSSION	21	Discuss the generalisability of the study results.	Discussion and Conclusion address global and Africa-specific implications while noting limits to country-specific, clade-specific, severity, and individual-level inference from aggregate surveillance data.
OTHER INFORMATION	22	Give the source of funding and the role of the funders for the present study and, if applicable, for the original study on which the present article is based.	Funding statement declares that the author received no specific funding for this work.
