# Supplementary material for: Global mpox outbreak dynamics during and after the 2024–2026 public health emergency
Source: PLoS One. 2026 Oct 21;21(10):e0350572. doi: 10.1371/journal.pone.0350572 (PMC13686470; doi:10.1371/journal.pone.0350572)
Supplement: Supplementary file 2 [file pone.0350572.s012.pdf]

Respond to Reviewers

PLOS ONE | Manuscript ID: **PONE-D-26-23896**

Date: 27 June 2026

To: The Academic Editor and Editorial Office, PLOS ONE

Re: Revised manuscript, "**Global mpox outbreak dynamics during and after the 2024-2026 public health emergency**"

Dear Academic Editor and PLOS ONE Editorial Office,

I thank the Academic Editor and all three Reviewers for their detailed, constructive, and expert engagement with this manuscript. The comments collectively identified important gaps in transparency, reproducibility, and interpretive caution, and I believe the revision addresses each concern substantively. I respond to every point below, organised by reviewer. Changes to the manuscript are indicated by section. A clean revised manuscript and a version with tracked changes are provided as separate files.

I respond in detail to each specific comment.

Responses to the Academic Editor

Editor comment 1: Novelty and added value must be highlighted better.

I have added an explicit novelty statement at the opening of the Discussion (first paragraph). I state that while recent syntheses have described the African epidemiology of the second PHEIC [refs 10, 11], the present analysis adds an integrated multi-domain framework spanning global and Africa-specific time-series modelling, DRC subnational burden, international travel-linked detection pathways, and the immediate post-emergency transition within a single structured analysis, including a 26-month global monthly series and a 112-week African weekly series with sensitivity analysis. I also added a one-to-two sentence positioning statement relative to references 10 and 11 specifically, as requested by Reviewer 3 Minor Point 8. (Revised Discussion, paragraph 1.)

Editor comment 2: Surveillance heterogeneity must be acknowledged explicitly and any circumventing approach detailed.

Surveillance heterogeneity is now treated as a central interpretive constraint rather than a secondary caveat throughout the revised manuscript. In the Methods (Data sources section), I added an explicit paragraph stating that dashboard data reflect information reported by national authorities and are subject to retrospective revision, differential reporting completeness, variable testing capacity, and differing case definitions across countries and time periods, and that these heterogeneities are treated as central interpretive limitations. In the Discussion, I added a dedicated paragraph on surveillance bias as a central constraint, explicitly naming testing capacity, case ascertainment, reporting practices, and surveillance intensity as factors affecting all comparisons in the analysis. In the Strengths and Limitations section, surveillance heterogeneity is now listed as the first and most fundamental limitation, with explicit acknowledgement that testing volumes and positivity rates were not available consistently in the public

datasets. (Revised Methods — Data sources; Revised Discussion — Surveillance bias paragraph; Revised Strengths and Limitations.)

Editor comment 3: Data acquisition, collection, and aggregation detail must be provided.

I have substantially expanded and refined the Data sources section of the Methods. The revised section identifies the WHO Global Mpox Trends dashboard as the primary analytic source; describes the core data streams used for global monthly trends, African weekly surveillance, DRC health-zone burden, demographic and epidemiological characteristics, and travel-linked detection analysis; specifies the April 2026 download date and component-specific analytic cutoffs; and clarifies that WHO situation reports and other official sources were used for contextual validation and policy-response timeline construction rather than to overwrite dashboard-derived quantitative values. This aligns the response with the final streamlined Methods text while preserving the requested provenance and aggregation detail. (Revised Methods — Data sources and study period.)

Editor comment 4: Manuscript must meet PLOS ONE style requirements.

I have verified section order, figure submission, table placement, ethics statement location, data availability section, competing interests, funding, and author contributions against the PLOS ONE style templates. All sections are compliant.

Editor comment 5: AI tools section must name specific tools used.

I have updated the Use of Artificial Intelligence Tools statement to name Claude (Anthropic) specifically, with a clarification of the scope of use. (Revised Use of Artificial Intelligence Tools section.)

Editor comment 6: Ethics statement must appear only in the Methods section.

The ethics statement remains in the Methods as a separate Ethical statement subsection. No duplicate ethics statement appears elsewhere. (Revised Methods — Ethical statement.)

Editor comment 7: Authors must be linked to affiliations.

The author affiliation is now listed on the title page as: Tambe Elvis Akem, Independent Public Health Consultant, Yaoundé, Cameroon. (Revised title page.)

Responses to Reviewer 1

Major Comment 1: Greater clinical context regarding clade-specific disease characteristics is needed

1a. Can the authors briefly summarise current evidence regarding clinical differences between clades?

I have added a paragraph to the Discussion summarising current evidence on clade-specific clinical context. I note that clade Ib outbreaks in eastern DRC have been characterised by broader age and sex distribution and predominantly non-sexual community and household transmission, compared with clade IIb, which has been concentrated in MSM networks with predominantly mild outcomes in immunocompetent adults in non-African settings. I also note that clade Ia has historically been reported in Central African settings with higher observed case fatality ratios, particularly among children, while cautioning that these estimates are also affected by surveillance intensity, case ascertainment, and care-access differences. (Revised Discussion — African burden, DRC subnational patterns, and clade biology, paragraph 3.)

1b. Are there available clinical datasets supporting severity differences between clades?

I acknowledge in the revised Discussion that population-level CFR estimates from aggregate surveillance data should not be used to draw conclusions about clade-specific virulence without individual-level clinical data. I do not have access to individual-level clinical datasets in this surveillance analysis, and I have been explicit about this limitation. (Revised Discussion — clade biology paragraph; Revised Strengths and Limitations.)

1c. Could differing age distributions or healthcare access explain CFR observations more than viral lineage?

Yes — and I have stated this explicitly. The revised manuscript removes the phrase "compatible with clade Ib predominance" from the CFR discussion and replaces it with a surveillance completeness explanation: the eastern provinces have been the focus of intensive outbreak response, generating greater ascertainment of mild and suspected cases; a larger suspected-case denominator mechanically lowers the calculated CFR without necessarily reflecting lower lethality. Age structure, healthcare access, testing strategy, and reporting completeness are all named as contributing factors. (Revised Results — DRC subnational burden; Revised Discussion — African burden paragraph 2.)

Major Comment 2: CFR interpretation requires stronger caution

2a. Do the authors have age-adjusted or severity-adjusted regional data?

Age-adjusted or severity-stratified data at the regional or provincial level were not available in the public dataset. I state this explicitly in the Strengths and Limitations section. (Revised Strengths and Limitations, point 6.)

2b. Were hospitalisation or severe disease indicators available at regional level?

No. Hospitalisation data in the WHO dashboard are available only as cumulative global proportions among cases with available data, not disaggregated by WHO region or DRC province. I acknowledge this in the Strengths and Limitations section. (Revised Strengths and Limitations.)

2c. Can the authors expand the discussion to better emphasise CFR limitations?

Yes. The eastern versus non-eastern DRC CFR contrast is now explicitly reframed in both Results and Discussion as a surveillance completeness artefact rather than an epidemiological severity signal. The mechanism — intensive outbreak attention generating larger suspected-case denominators — is explained. Phase-level CFR comparisons in Table 1 carry an explicit note that CFRs are sensitive to differential testing intensity and should not be interpreted as true lethality estimates or compared directly across phases. Monthly CFR values are noted to be volatile given small death counts and are not reported month-by-month. (Revised Results — DRC subnational burden; Revised Results — Global epidemic trajectory; Revised Discussion.)

Major Comment 3: Community transmission terminology should be clarified

3a. Were authors able to distinguish isolated secondary cases from sustained local transmission?

No. The WHO importation tracking dataset records onward cases as a binary indicator without providing the number of subsequent cases or the duration of local spread. The revised manuscript explicitly states that the secondary transmission (ST) indicator captures events with a single secondary case as well as those with multiple secondary cases and does not distinguish isolated transmission from sustained local spread. ST is presented as a documented surveillance outcome rather than a measure of transmissibility. (Revised

Methods — Travel-linked detection and secondary-transmission analysis; Revised Results — International clade importation and documented secondary transmission.)

3b. Could alternative wording such as "documented secondary transmission" be considered?

Yes. I have adopted this terminology throughout the revised manuscript. "Community transmission" and its abbreviation "CT" have been replaced with "documented secondary transmission" and "ST" in all sections, including the Methods, Results, Table 3, and associated table notes. (Revised manuscript throughout.)

Major Comment 4: Interpretation of travel-linked pathways should be further clarified

4a. Were travel pathways derived solely from reported travel history, or were exposure investigations incorporated?

Travel pathways are derived from the reported place of origin in the WHO clade I MPXV importation/travel-linked detection dashboard export, which reflects reported travel history of the index case. Detailed exposure investigations were not incorporated; these were not available in the public dataset. I now state this explicitly in the Methods. (Revised Methods — Travel-linked detection and secondary-transmission analysis.)

4b. Was any genomic epidemiology available to support travel-associated linkage?

Genomic data were not available at the level needed to confirm travel-associated linkage in this analysis. I acknowledge this as a limitation. (Revised Strengths and Limitations; Revised Discussion — International spread paragraph.)

4c. Could aviation-hub effects have influenced frequency of reported linkage?

Yes, and I now state this explicitly in both the Results and Discussion. The prominence of the UAE as the most frequently reported place of origin may partly reflect its role as a major international aviation hub rather than local epidemiological amplification, and transit-country effects cannot be distinguished from source-country exposure without detailed contact investigation or genomic data. (Revised Results — International clade importation and documented secondary transmission; Revised Discussion — International spread paragraph.)

Major Comment 5: The manuscript occasionally risks implying causal relationships from temporal associations

5a. Were sensitivity analyses performed using alternative breakpoints?

Yes. I have added a revision sensitivity analysis shifting the April 2025 breakpoint by minus one, plus one, and plus two months. Across all four specifications, the post-breakpoint slope remained consistently below 0.82 and highly significant ($p < 0.001$), supporting robustness to the exact breakpoint location. These results are reported in the Results (Global epidemic trajectory section) and in a new Breakpoint Sensitivity section of the revised Table 2. (Revised Results — Global epidemic trajectory; Revised Table 2.)

5b. Were lag periods considered for policy-response effects?

Lag effects of policy interventions cannot be modelled in the two-breakpoint ITS framework used, which captures immediate step changes and slope changes at prespecified time points. I acknowledge in the Methods that autocorrelation was diagnosed in some model residuals but was not corrected in the primary analysis, which used the default covariance estimator, and that model estimates reflect temporal associations rather than causal effects. (Revised Methods — Statistical analysis.)

5c. Could observed changes reflect the natural epidemic curve?

Yes — and I now state this directly in the Results and Discussion. The April 2025 breakpoint falls one month before the May 2025 global epidemic peak, so the decline identified in the model largely reflects the natural post-peak descent of the epidemic curve rather than any effect of the SPRP update. I state this explicitly wherever the April 2025 estimates are reported, and repeat it in the Discussion and Limitations. (Revised Results — Global epidemic trajectory; Revised Discussion — Epidemic trajectory paragraph; Revised Strengths and Limitations, point 7.)

Major Comment 6: More discussion on surveillance bias would strengthen interpretation

6a. Were testing volumes or positivity rates available?

Testing volumes and positivity rates were not available consistently in the public datasets used for this analysis. I state this explicitly in the Strengths and Limitations section. (Revised Strengths and Limitations, point 1.)

6b. Did any countries undergo major surveillance changes during the study period?

Major surveillance system changes at the country level cannot be identified from the available aggregate data. I acknowledge this in the Limitations. (Revised Strengths and Limitations, point 1.)

6c. Can the authors discuss how surveillance intensity may have influenced apparent epidemic growth?

Yes. A dedicated paragraph on surveillance bias has been added to the Discussion, stating that changes in reported case rates reflect both transmission dynamics and surveillance intensity, that the immediate rise at the PHEIC declaration likely reflects surveillance intensification alongside transmission acceleration, and that the subsequent decline may reflect both genuine epidemic decline and surveillance normalisation during a prolonged emergency. (Revised Discussion — Surveillance bias as a central interpretive constraint paragraph.)

Major Comment 7: More clinical interpretation of demographic findings would be valuable

7a. Can the authors better contextualise the observed rise in MSM-associated and travel-associated reporting?

Yes. The revised demographic Results section and Discussion both contextualise the observed shifts in proportions. The rising proportions of reported MSM status and sexual transmission in the recent period likely reflect continued concentration of reporting in well-networked populations with better diagnostic access in non-African settings, rather than a true increase in risk or shift in transmission biology. The higher reported travel-history proportion reflects the importation-intensive character of recent clade Ib detections outside Africa. (Revised Results — Contextual global demographic profile; Revised Discussion — International spread paragraph.)

7b. Could missing data or reporting bias partly explain these findings?

Yes, and I now state this explicitly. The recent-period case subset represents a small and non-random fraction of the cumulative total, with different reporting completeness, surveillance coverage, and geographic mix. These observations are presented descriptively without inferential p-values in the revised manuscript. The detailed tabulated comparison has been moved to Supplementary Table S4. (Revised Results — Contextual global demographic profile; Revised Strengths and Limitations.)

Major comment 8: statistical-analysis comment

I thank the reviewer for this balanced assessment of the statistical approach. I have retained the negative binomial segmented regression framework with log-population offsets because it is appropriate for the overdispersed surveillance count data used in this study. I have also strengthened the methodological explanation in the revised Methods by clarifying the rationale for breakpoint selection, the use of overdispersion diagnostics, the assessment of residual autocorrelation using the Durbin-Watson statistic on Pearson residuals, and the interpretation of model outputs as reported case-rate ratios rather than true infection incidence.

Regarding breakpoint selection, the PHEIC declaration and April 2025 SPRP update were prespecified policy-response milestones and were not selected post hoc to optimise model fit. The April 2025 breakpoint was additionally tested in sensitivity analyses shifted by minus one, plus one, and plus two months; the post-breakpoint slope remained consistently below 1.0 and statistically significant across specifications. The March 2025 ecological comparison has been retained only as an exploratory supplementary descriptive analysis, not as a causal breakpoint.

I have also strengthened interpretive caution around ecological and temporal associations. The revised manuscript states that observed changes around policy milestones and the March 2025 analytic boundary may reflect epidemic timing, surveillance intensity, testing practices, and reporting changes, and should not be interpreted as causal effects. The logistic regression of documented secondary transmission is now labelled exploratory throughout, with explicit acknowledgement of the small number of secondary-transmission events, limited events-per-variable ratio, wide confidence intervals, and complete separation for AMRO. Conclusions from this model have therefore been kept conservative.

Minor Comments

Minor Comment 1: Dense statistical text in some sections.

I have shortened several paragraphs in the Results and Discussion, moved secondary analyses to Supplementary Information, and removed the event-study section from the main Table 2. The revised manuscript is more concise.

Minor Comment 2: Summary figure showing epidemic phases, clade predominance, geographic spread, and policy milestones.

Supplementary Figure S3 (integrated epidemic and policy-response timeline) illustrates major epidemic phases and policy milestones across the study period. I have updated the S3 Fig reference in the Results to draw readers' attention to this figure explicitly. A new multi-panel summary figure combining all four domains was considered but would require additional preparation beyond the current revision scope; the existing S3 Fig addresses the policy and phase overview component.

Minor Comment 3: Define abbreviations at first use consistently.

All abbreviations are now defined at first use in the main text. Abbreviation definitions have also been added independently within table notes and figure legends so that tables and figures are self-contained. (Revised table notes for Tables 1, 2, and 3; revised figure legends for Figs 1, 2, 3, and S1 Fig.)

Minor Comment 4: DRC hotspot classification methodology — brief explanation in simpler terms.

The hotspot classification methodology is now described in plain language in the revised DRC subnational burden Results section: health zones were classified as persistent hotspot, emerging hotspot, historical

hotspot, or low-burden using the 75th percentile of cumulative and recent 12-month case counts across all 200 zones, with percentile thresholds defined prior to analysis. (Revised Results — DRC subnational burden.)

Minor Comment 5: Clinical implications for clinicians outside endemic regions.

I have added a sentence in the Discussion contextualising findings for non-endemic clinicians: clinicians outside endemic regions should be aware that travel history to sub-Saharan Africa or East Africa and MSM status are the predominant reported risk factors in non-African settings, while presentations in African settings follow fundamentally different demographic and transmission profiles characterised by broader age and sex distributions, household contacts, and non-sexual transmission routes. (Revised Discussion — International spread and secondary transmission paragraph.)

Responses to Reviewer 2

Major Comment 1: Data collection and curation must be described in greater detail

I have substantially revised the Data sources section of the Methods to address this concern. The revised section identifies the WHO Global Mpox Trends dashboard as the primary analytic database, states that all WHO dashboard datasets were downloaded in April 2026, and specifies the component-specific analytic cutoffs: global monthly confirmed case analyses and travel-linked detection analyses used the 28 February 2026 primary analytic cutoff; African weekly surveillance data were current through 22 March 2026 but were truncated to 28 February 2026 for the primary time-series analysis; DRC subnational health-zone burden used data current to mid-March 2026; and demographic and epidemiological characteristic data were available through March 2026. The source hierarchy is stated explicitly: the WHO dashboard export is the primary quantitative source, while situation reports and other official sources are used for contextual validation and policy-response timeline construction. For travel-linked detection records, the revised manuscript clarifies that records derive from the WHO dashboard export and were filtered to the primary cutoff to yield 163 records. (Revised Methods — Data sources and study period.)

Major Comment 2: Surveillance limitations not sufficiently incorporated into interpretation

This concern is now central to the revised manuscript rather than peripheral. Surveillance heterogeneity is named in the Methods as affecting comparability across regions and time periods and is treated as a central interpretive limitation. In the Discussion, a dedicated paragraph states that all findings are based on aggregate surveillance data with heterogeneous practices and that changes in reported case rates reflect both transmission dynamics and surveillance intensity, which cannot be disentangled. In the Strengths and Limitations, surveillance heterogeneity is listed as the first and most fundamental limitation, with explicit acknowledgement that testing volumes and positivity rates were not available consistently. (Revised Methods; Revised Discussion — Surveillance bias paragraph; Revised Strengths and Limitations.)

Major Comment 3: Clarify the added value of the study

As described in the response to the Editor, I have added an explicit novelty statement at the opening of the Discussion. I also added a positioning statement relative to references 10 and 11 specifically. (Revised Discussion, paragraph 1.)

Major Comment 4: Consider closer engagement with data providers

I appreciate this recommendation and acknowledge it as a valuable direction for future work. The present analysis is based on publicly available surveillance data maintained by WHO and partner agencies. Closer engagement with data providers and reporting systems could in future help to better characterise known limitations, reporting artefacts, and changes in surveillance systems over time. In addition, with permission, I have added an acknowledgement for technical review of an earlier version of the manuscript; responsibility for the analysis, interpretation, conclusions, and final content remains solely with the author.

Major Comment 5: Simplify and focus the manuscript

I have made several structural simplifications in the revised manuscript: (i) the event-study windows have been moved from Table 2 to Supplementary Table S1; (ii) the March 2025 ecological comparison has been moved from the main Results to Supplementary Table S3 and S4 Fig, with only a brief hypothesis-generating note in the main text; (iii) the detailed demographic comparisons have been moved to Supplementary Table S4 and are presented descriptively without inferential p-values; and (iv) the financing context has been retained only as brief contextual interpretation, explicitly stating that the ecological comparison is not evidence of a financing effect on mpox trajectory. (Revised Table 2; Revised Results — Demographics; Revised Results — March 2025 note; new Supplementary Tables S1, S3, S4.)

Major Comment 6: Reconsider some of the statistical analyses

I have retained the segmented negative binomial regression and interrupted time-series framework as the primary analytical approach, as this is appropriate for the research question and the available overdispersed count data (Poisson Pearson dispersion: 104.76 for the global monthly model; 206.21 for the African weekly model). I have, however, made the following changes in response to this comment: (i) I moved the event-study from the main manuscript to Supplementary Table S1; (ii) I explicitly label the logistic regression for secondary transmission as exploratory throughout the manuscript; (iii) I acknowledge the small events-per-variable ratio and wide confidence intervals wherever these results are reported; and (iv) I removed the regional panel model from the Results, replacing it with descriptive language about AFRO's share of global burden. (Revised Results throughout; Revised Table 2.)

Responses to Reviewer 3

Major Point 1: Reconcile global and DRC case totals

I thank Reviewer 3 for identifying this critical issue precisely. The apparent discrepancy arises because the two totals use fundamentally different case definitions and different reporting systems (though reported on a single dashboard, the WHO global dashboard): the global total of 82,738 represents confirmed cases only; the DRC subnational total of 144,035 represents total reported cases (suspected and clinically compatible, plus confirmed) from DRC national subnational health-zone surveillance, which aggregates all notified cases at the health-zone level without a confirmed-case breakdown. These are not directly comparable.

In the revised manuscript: (a) the case definition is stated explicitly for every total reported — in the Abstract, Methods, Results, and Limitations; (b) the non-comparability is stated directly wherever both figures appear; (c) I confirm that no derived quantities in this analysis mix the two case definitions; (d) the Abstract now includes both the death count (2,047) and the case definition for the DRC subnational figures. Exact data cutoff dates and case definitions for each analytical component are described in the expanded Data sources section. (Revised Abstract; Revised Methods — Data sources; Revised Results — DRC subnational burden; Revised Strengths and Limitations.)

Major Point 2: Do not anchor severity inferences on the DRC CFRs

I fully accept this point. The revised manuscript explicitly reframes the eastern versus non-eastern DRC CFR contrast as a surveillance completeness artefact rather than an epidemiological severity signal in both Results and Discussion. I explain the mechanism: intensive outbreak response generates greater ascertainment of mild and suspected cases in eastern provinces, producing a larger suspected-case denominator that mechanically lowers the calculated CFR. The phrase "compatible with clade Ib predominance" has been removed from the Discussion. I state clearly that this comparison cannot be interpreted as evidence of clade-specific virulence differences. The DRC subnational CFRs are now described as ratios of reported deaths to total reported health-zone cases, not as lethality estimates. (Revised Results — DRC subnational burden; Revised Discussion — African burden paragraph 2.)

Major Point 3: State plainly that the April 2025 breakpoint coincides with the epidemic peak

I now state this directly and prominently wherever the April 2025 breakpoint is discussed. In the Results (Global epidemic trajectory section), I state that the April 2025 breakpoint falls one month before the May 2025 global case peak and that the subsequent decline identified in the model therefore largely reflects the natural post-peak descent of the epidemic curve rather than any effect of the SPRP update. This statement is repeated in the Discussion and in the Strengths and Limitations. Additionally, I have added a revision sensitivity analysis shifting the April 2025 breakpoint by minus one, plus one, and plus two months; the post-breakpoint slope remained consistently below 0.82 and highly significant across all specifications. (Revised Results — Global epidemic trajectory; Revised Discussion; Revised Strengths and Limitations, point 7; Revised Table 2 — Breakpoint Sensitivity section.)

Major Point 4: Resolve the "presented-then-disclaimed" pattern

I have resolved this pattern through structural changes:

(i) The eight-week event-study windows and percentage changes have been moved from Table 2 to Supplementary Table S1, where they are presented as descriptive trajectory summaries without effect-style language, causal interpretation, or inferential emphasis.

(ii) The March 2025 ecological comparison has been moved from a full Results analysis to Supplementary Table S3 and S4 Fig, with only a brief note in the main Results and a concise financing-context paragraph in the Discussion. Both state that the comparison is hypothesis-generating and should not be interpreted as evidence of a financing effect on epidemic trajectory.

(iii) The demographic comparisons are presented descriptively in the main text without inferential p-values. Full tabulated comparisons are provided in Supplementary Table S4, with a prominent note that these data are cumulative from 2022 and do not represent the primary study period.

(Revised Table 2; Revised Results — Demographics; Revised Results — March 2025 note; Revised Discussion — Financing context; new Supplementary Tables S1, S3, S4.)

Major Point 5: Expand discussion of surveillance and reporting bias

A dedicated surveillance bias paragraph has been added to the Discussion, and surveillance heterogeneity is now the first and most fundamental limitation in the Strengths and Limitations section. The revised Discussion explicitly connects surveillance bias to: (i) the global/DRC denominator reconciliation; (ii) the CFR artefacts in eastern DRC; and (iii) regional secondary transmission rate differences, where I note that regional differences in documented ST rates likely reflect testing intensity, contact-tracing capacity, and surveillance completeness rather than a single biological mechanism. (Revised Discussion — Surveillance bias paragraph; Revised Strengths and Limitations, point 1.)

Major Point 6: Improve reproducibility and deposit analysis code

During revision, the analysis code was rewritten and validated against the raw source CSV files and the curated master dataset to ensure reproducibility of the principal numerical results, tables, and figures. This process identified minor numerical discrepancies between the originally submitted model outputs and the clean reproducible code, arising from implementation-level differences in model fitting and region-mapping standardisation. I updated affected reported case-rate ratios, confidence intervals, p-values, and associated narrative text to match the verified reproducible outputs. The direction of all principal findings was preserved, with one exception: the within-PHEIC global monthly slope is now described as a non-significant trend toward deceleration (CRR 0.933, $p = 0.066$) rather than a statistically significant decline.

The annotated Python reproducibility package, requirements file, README, curated source datasets, validation checks, and generated tables and figures are publicly deposited in the GitHub repository named in the Data Availability Statement. Exact data download dates and analytic cutoffs are specified in the Data sources section of the Methods. (Revised Data Availability Statement; Revised Methods — Data sources and Statistical analysis; Revised Table 2.)

Major Point 7: Strengthen provenance and bias treatment for the travel-linked detection/ST dataset

The travel-linked detection dataset is now described explicitly as a WHO-maintained dashboard export of clade I MPXV importation or travel-linked detection records. The analysis uses the 163 records within the primary analytic cutoff of 28 February 2026; records dated after the cutoff were retained in the supporting source dataset but excluded from primary analyses. The revised manuscript clarifies that the author did not independently adjudicate event provenance beyond applying the prespecified cutoff and using the reported place-of-origin field. The logistic regression model for documented secondary transmission (ST) is labelled exploratory throughout the manuscript, not only at first mention. (Revised Methods — Travel-linked detection and secondary-transmission analysis; Revised Results — International clade importation; Revised Table 3.)

Minor Point 1: IRR terminology should be changed to a distinct term

I have replaced "incidence rate ratio (IRR)" with "reported case-rate ratio (CRR)" throughout the manuscript, including all text and Table 2. CRR is defined at first use in the Methods (Statistical analysis section) and in the Abstract. (Revised Abstract; Revised Methods; Revised Table 2; Revised Results throughout.)

Minor Point 2: Underspecified statistics

I have added the following to the Methods (Statistical analysis section): (i) overdispersion diagnostics are described as the basis for retaining negative binomial rather than Poisson models; (ii) the NB dispersion parameter alpha and the rationale for NB over Poisson are described; (iii) Python libraries and versions are specified; (iv) residual autocorrelation was assessed using the Durbin-Watson statistic on Pearson residuals and is noted as a diagnostic finding; the primary analysis used the default covariance estimator without HAC correction; and (v) the event-study percentage changes are now presented as descriptive summaries in Supplementary Table S1 without inferential emphasis. (Revised Methods — Statistical analysis.)

Minor Point 3: Multiple comparisons

I have added a statement in the Methods (logistic regression paragraph) noting that multiple comparisons were made across destination regions and origin groups without formal adjustment, and that borderline p-values should be treated with caution. This statement is repeated in the Results (importation section). (Revised Methods; Revised Results — International clade importation.)

Minor Point 4: Shifting analytic cutoffs

Exact data cutoff dates and case definitions for each analytical component are now described in the expanded Data sources section of the Methods. I clarify that global monthly confirmed case analyses and importation/travel-linked detection analyses use the 28 February 2026 primary cutoff; African weekly surveillance data were current through 22 March 2026 but the primary model was truncated to 28 February 2026; DRC subnational burden uses mid-March 2026 data; and demographic data were available through March 2026. (Revised Methods — Data sources.)

Minor Point 5: Monthly CFR volatility

I have added a note in the Results (Global epidemic trajectory section) stating that monthly CFR estimates are volatile because of the small number of deaths relative to cases in individual months and should be interpreted at the phase level rather than month by month. Phase-level CFRs are presented in Table 1. (Revised Results — Global epidemic trajectory, paragraph 1.)

Minor Point 6: Define abbreviations at first appearance in tables and figures

Abbreviation definitions have been added independently within table notes and figure legends so that all tables and figures are self-contained. (Revised Table 1, Table 2, and Table 3 notes; revised Fig 1, Fig 2, Fig 3, and S1–S4 Fig legends.)

Minor Point 7: Travel-linked detections versus true importations

I have added an explicit distinction in the Methods and Results. The Methods state that the dataset captures importation or travel-linked detection events, representing documented detections rather than all international importations. The Results explicitly note that place of origin reflects reported travel history and does not confirm the site of infection or transmission, and that transit-country and aviation-hub effects may influence the frequency with which certain countries appear as apparent source nodes. The UAE statement reads that its prominence as reported origin "may partly reflect its role as a major international aviation hub rather than local epidemiological amplification." (Revised Methods; Revised Results — International clade importation.)

Minor Point 8: Positioning relative to existing syntheses

I have added a positioning statement relative to references 10 (Kazadi et al. 2026) and 11 (Ndembi et al. 2025) at the opening of the Discussion. (Revised Discussion, paragraph 1.)

Minor Point 9: Supporting files not assessable in review package

The revised submission includes all supporting files as accessible attachments. S1 Checklist, S1–S4 Tables, and S1–S4 Figs all submitted as accessible files. All figure TIFFs are confirmed at 600 DPI with LZW compression.

Minor Point 10: Figure resolution

All main text figures (Fig 1, Fig 2, Fig 3) and supplementary figures (S1–S4 Fig) are submitted as TIFF files at 600 DPI with LZW compression, all under 10 MB. (Confirmed figure files at 600 DPI.)

Submission package and compliance

The revised submission includes a clean manuscript file, a marked-up manuscript file, a detailed response-to-reviewers document letter, revised main and supplementary figures, revised supplementary tables, the STROBE checklist, and a reproducibility package with source data and Python code.

The revision retains the study's original public-health objective while improving technical transparency and interpretive caution. The manuscript includes updated Data Availability, Funding, Competing Interests, Author Contributions, Ethics, Artificial Intelligence Tools statements, and acknowledgement.

I believe the revised manuscript now addresses the editor's and reviewers' concerns and meets PLOS ONE's expectations for transparent reporting, data availability, reproducibility, and scientific soundness.

Thank you again for your consideration of this revised manuscript.

Sincerely,

Tambe Elvis Akem

Independent Public Health Consultant

Yaounde, Cameroon

Correspondence: tambeelvis2008@yahoo.com

ORCID: https://orcid.org/0009-0007-5699-8999
