# Supplementary material for: Global mpox outbreak dynamics during and after the 2024–2026 public health emergency
Source: PLoS One. 2026 Oct 21;21(10):e0350572. doi: 10.1371/journal.pone.0350572 (PMC13686470; doi:10.1371/journal.pone.0350572)
Supplement: Supplementary file 3 [file pone.0350572.s002.docx]

**S1 Table.** Descriptive pre-/post-window changes in reported confirmed mpox cases around selected policy milestones

| **Milestone** | **Date** | **Global monthly window** | **Global pre-window mean** | **Global post-window mean** | **Change (%)** | **Africa weekly window** | **Africa pre-window mean** | **Africa post-window mean** | **Change (%)** |
| --- | --- | --- | --- | --- | --- | --- | --- | --- | --- |
| WHO PHEIC declaration | 14 Aug 2024 | Jun–Jul 2024 vs Sep–Oct 2024 | 1,840 | 3,570 | +94.0% | 8 weeks before vs 8 weeks after | 264 | 426 | +61.2% |
| April 2025 SPRP update | 17 Apr 2025 | Feb–Mar 2025 vs May–Jun 2025 | 4,595 | 6,767 | +47.3% | 8 weeks before vs 8 weeks after | 885 | 1,421 | +60.6% |
| WHO PHEIC termination | 5 Sep 2025 | Jul–Aug 2025 vs Oct–Nov 2025 | 4,651 | 2,899 | −37.7% | 8 weeks before vs 8 weeks after | 805 | 648 | −19.4% |
| Africa CDC PHECS lifted | 22 Jan 2026 | Nov–Dec 2025 vs Feb 2026 | 2,060 | 1,184 | −42.5% | 8 weeks before vs 5 available weeks after | 240 | 218 | −9.2% |

**Note:** Values are mean reported confirmed case counts in pre- and post-milestone windows. For global monthly data, 8 weeks were approximated using two full calendar months before and after each milestone, excluding the milestone month where possible. For African weekly surveillance, exact week-ending dates were used: eight weeks before and eight weeks after each milestone. For the Africa CDC PHECS lifting milestone, the post-window was truncated by the 28 February 2026 primary analytic cutoff; only one full post-event month (February 2026) was available for global monthly data and five post-event weeks were available for African weekly data. No p-values are reported; these summaries are descriptive and should not be interpreted as causal effects of policy milestones. PHEIC, WHO Public Health Emergency of International Concern; PHECS, Africa CDC Public Health Emergency of Continental Security; SPRP, WHO Strategic Preparedness and Response Plan.
