# Supplementary material for: Global mpox outbreak dynamics during and after the 2024–2026 public health emergency
Source: PLoS One. 2026 Oct 21;21(10):e0350572. doi: 10.1371/journal.pone.0350572 (PMC13686470; doi:10.1371/journal.pone.0350572)
Supplement: Supplementary file 4 [file pone.0350572.s003.docx]

**S2 Table.** Top 20 DRC health zones by cumulative reported mpox burden and hotspot typology, onset of outbreak to mid-March 2026

| **Rank** | **Health zone** | **Province** | **Total reported cases (n)** | **Share of national reported burden (%)** | **Total reported deaths (n)** | **Observed zone CFR (%)** | **Recent reported cases (n)** | **Eastern province** | **Typology** |
| --- | --- | --- | --- | --- | --- | --- | --- | --- | --- |
| 1 | Miti-Murrhesa | Sud-Kivu | 9,695 | 6.7 | 11 | 0.11 | 220 | Yes | Persistent hotspot (PH) |
| 2 | Nyangezi | Sud-Kivu | 5,600 | 3.9 | 2 | 0.04 | 256 | Yes | Persistent hotspot (PH) |
| 3 | Karisimbi | Nord Kivu | 5,032 | 3.5 | 0 | 0.00 | 176 | Yes | Persistent hotspot (PH) |
| 4 | Budjala | Sud-Ubangi | 3,845 | 2.7 | 23 | 0.60 | 44 | No | Persistent hotspot (PH) |
| 5 | Ikela | Tshuapa | 3,682 | 2.6 | 26 | 0.71 | 153 | No | Persistent hotspot (PH) |
| 6 | Lingomo | Tshuapa | 3,673 | 2.6 | 181 | 4.93 | 201 | No | Persistent hotspot (PH) |
| 7 | Nyiragongo | Nord Kivu | 3,033 | 2.1 | 0 | 0.00 | 85 | Yes | Persistent hotspot (PH) |
| 8 | Lotumbe | Equateur | 2,894 | 2.0 | 224 | 7.74 | 25 | No | Historical hotspot (HH) |
| 9 | Dikungu | Sankuru | 2,770 | 1.9 | 54 | 1.95 | 130 | No | Persistent hotspot (PH) |
| 10 | Uvira | Sud-Kivu | 2,733 | 1.9 | 7 | 0.26 | 49 | Yes | Persistent hotspot (PH) |
| 11 | Yalimbongo | Tshopo | 2,696 | 1.9 | 210 | 7.79 | 79 | No | Persistent hotspot (PH) |
| 12 | Monga | Bas Uele | 2,576 | 1.8 | 25 | 0.97 | 37 | No | Persistent hotspot (PH) |
| 13 | Wembo-Nyama | Sankuru | 2,570 | 1.8 | 1 | 0.04 | 94 | No | Persistent hotspot (PH) |
| 14 | Nyantende | Sud-Kivu | 2,528 | 1.8 | 1 | 0.04 | 136 | Yes | Persistent hotspot (PH) |
| 15 | Bokungu | Tshuapa | 2,335 | 1.6 | 56 | 2.40 | 25 | No | Historical hotspot (HH) |
| 16 | Tshumbe | Sankuru | 2,016 | 1.4 | 7 | 0.35 | 139 | No | Persistent hotspot (PH) |
| 17 | Tshudi-Loto | Sankuru | 1,969 | 1.4 | 28 | 1.42 | 185 | No | Persistent hotspot (PH) |
| 18 | Lusambo | Sankuru | 1,944 | 1.3 | 17 | 0.87 | 133 | No | Persistent hotspot (PH) |
| 19 | Minova | Sud-Kivu | 1,881 | 1.3 | 2 | 0.11 | 76 | Yes | Persistent hotspot (PH) |
| 20 | Katako-Kombe | Sankuru | 1,850 | 1.3 | 13 | 0.70 | 50 | No | Persistent hotspot (PH) |

**Note:** Total reported cases represent aggregates of clinically compatible, suspected, and confirmed cases at health-zone level as reported to the DRC national surveillance system; no confirmed/suspected breakdown is available at this level, and observed zone CFRs should not be interpreted as true lethality estimates. Share (%) represents each zone’s cumulative reported cases as a percentage of the 200-zone national total (n = 144,035). Recent reported cases are cases reported in the 12-month window before the mid-March 2026 data cutoff. Eastern province indicates that the health zone is located within one of seven eastern provinces (Sud-Kivu, Nord Kivu, Maniema, Tanganyika, Haut Lomami, Haut Katanga, and Lualaba), used as a geographic proxy for clade Ib distribution because zone-level clade confirmation data were unavailable. Typology was assigned using 75th percentile thresholds for cumulative reported cases (≥992 cases) and recent 12-month reported cases (≥36 cases) across all 200 zones. PH, persistent hotspot: high cumulative burden and high recent burden; EH, emerging hotspot: lower cumulative burden but high recent burden; HH, historical hotspot: high cumulative burden but lower recent burden; LB, low-burden: lower cumulative burden and lower recent burden. Across all 200 zones, PH = 39, EH = 12, HH = 12, and LB = 137.
