# Supplementary material for: Global mpox outbreak dynamics during and after the 2024–2026 public health emergency
Source: PLoS One. 2026 Oct 21;21(10):e0350572. doi: 10.1371/journal.pone.0350572 (PMC13686470; doi:10.1371/journal.pone.0350572)
Supplement: Supplementary file 5 [file pone.0350572.s004.docx]

**S3 Table.** Exploratory ecological comparison of reported mpox indicators before and after the March 2025 analytic boundary

| **Indicator** | **Pre-boundary median (IQR)** | **Post-boundary median (IQR)** | **Change** | **p-value** |
| --- | --- | --- | --- | --- |
| Global monthly confirmed cases | 2,254 (IQR 1,174–3,862) | 4,471 (IQR 2,350–4,912) | +98.4% | 0.033 |
| Global monthly confirmed deaths | 6 (IQR 5–11) | 14 (IQR 8–21) | +8 deaths/month | 0.075 |
| Countries reporting (monthly) | 45 (IQR 37–50) | 51.5 (IQR 49–53) | +14.4% | 0.010 |
| Africa weekly confirmed cases | 306 (IQR 100–631) | 736 (IQR 297–932) | +140.4% | <0.001 |
| Africa weekly confirmed deaths | 1 (IQR 0–2) | 3 (IQR 1–5) | +2 deaths/week | <0.001 |

**Note:** The March 2025 boundary is an operational analytic reference point, not a causal breakpoint. All findings are exploratory and hypothesis-generating only and should not be interpreted as evidence of a financing effect or any other specific causal mechanism. The post-boundary period overlaps with the approach to the May 2025 global epidemic peak; observed increases may therefore reflect epidemic timing as much as any change in surveillance or response conditions. The pre-boundary period was January 2024 to February 2025 for global monthly indicators and 60 African surveillance weeks ending before March 2025. The post-boundary period was March 2025 to February 2026 for global monthly indicators and 52 African surveillance weeks through the 28 February 2026 primary analytic cutoff. Global monthly confirmed cases and deaths are WHO Global Mpox Trends dashboard confirmed-case totals. Countries reporting refers to countries with at least one confirmed case in the calendar month. Comparisons used the two-sided Mann-Whitney U test. ns, not significant.

(a) Africa weekly confirmed deaths are deaths reported among confirmed cases only; deaths among suspected cases, which substantially exceed confirmed-case deaths in the African surveillance series, are not included.
