# Supplementary material for: Global mpox outbreak dynamics during and after the 2024–2026 public health emergency
Source: PLoS One. 2026 Oct 21;21(10):e0350572. doi: 10.1371/journal.pone.0350572 (PMC13686470; doi:10.1371/journal.pone.0350572)
Supplement: Supplementary file 6 [file pone.0350572.s005.docx]

**S4 Table.** Demographic and epidemiological characteristics, cumulative and recent period comparisons, 2022-March 2026.

**Part A. Sex and age distribution (cumulative confirmed cases with age/sex data, n = 99,463)**

| **Category** | **n** | **% of confirmed cases with age/sex data** |
| --- | --- | --- |
| **Sex** | | |
| Male | 96,199 | 96.7% |
| Female | 3,264 | 3.3% |
| **Age group (years)** | | |
| 0–9 | 346 | 0.3% |
| 10–19 | 1,500 | 1.5% |
| 20–29 | 26,661 | 26.8% |
| 30–39 | 41,019 | 41.2% |
| 40–49 | 20,730 | 20.8% |
| 50–59 | 7,407 | 7.4% |
| 60–69 | 1,565 | 1.6% |
| ≥70 | 235 | 0.2% |
| **Selected subgroup summaries** | | |
| *Males aged 20–49 years (combined)* | *85,876* | *86.3%* |
| *Males aged 30–39 years (a)* | *40,112* | *40.3%* |

**Part B. Epidemiological and clinical characteristics (cumulative vs recent 12-month period)**

| **Variable** | **Cumulative (2022–Mar 2026) n / N** | **%** | **Recent 12-month period n / N** | **%** |
| --- | --- | --- | --- | --- |
| Men who have sex with men (MSM) | 34,524 / 39,541 | 87.3% | 2,042 / 2,214 | 92.2% |
| Persons living with HIV (PLHIV) | 20,163 / 39,726 | 50.8% | 699 / 1,718 | 40.7% |
| Health worker | 1,612 / 42,279 | 3.8% | 38 / 1,391 | 2.7% |
| Travel history reported | 5,567 / 32,165 | 17.3% | 514 / 1,997 | 25.7% |
| Sexual transmission reported | 23,129 / 25,980 | 89.0% | 2,124 / 2,213 | 96.0% |
| Hospitalised | 7,121 / 80,562 | 8.8% | 249 / 2,769 | 9.0% |
| ICU admission | 52 / 12,547 | 0.4% | 2 / 870 | 0.2% |
| Died | 201 / 64,895 | 0.3% | 0 / 2,284 | 0.0% |

**Note:** These data are derived from aggregated global WHO surveillance data with available demographic, epidemiological, and clinical-characteristic information. They span the 2022 outbreak through March 2026 and cannot be restricted to the January 2024 to February 2026 primary study period. All figures refer to confirmed cases with available data for the relevant variable. The cumulative dataset is heavily weighted by the 2022–2023 clade IIb outbreak concentrated among adult men in non-African settings and should not be generalised to African clade I or clade Ib outbreaks. The recent 12-month period reflects the most recent dashboard window ending in March 2026. Age and sex data were available for 99,463 confirmed cases cumulatively; no separate recent-period age-sex breakdown was available. Epidemiological and clinical characteristics use variable-specific denominators; proportions represent cases with the attribute divided by cases with available data for that variable. Observed differences between cumulative and recent-period proportions should be interpreted cautiously because denominators, reporting completeness, surveillance coverage, and geographic mix differ across variables and periods.

**(a)** Males aged 30–39 years = 40,112 (40.3% of all 99,463 confirmed cases with age/sex data). All cases aged 30–39 (both sexes combined) = 41,019 (41.2%). The figure 40,112 is cited in the manuscript text as the largest single age-sex category. n/N in Part B: n = cases with the attribute; N = cases with available data for that variable; denominator varies across variables.
