# Supplementary material for: Global mpox outbreak dynamics during and after the 2024–2026 public health emergency
Source: PLoS One. 2026 Oct 21;21(10):e0350572. doi: 10.1371/journal.pone.0350572 (PMC13686470; doi:10.1371/journal.pone.0350572)
Supplement: Supplementary file 7 [file pone.0350572.s010.docx]

**Overall Assessment**

This manuscript addresses an important and highly relevant global infectious disease issue. The study provides a broad and well-structured epidemiological overview of mpox dynamics during and after the second WHO Public Health Emergency of International Concern (PHEIC), with useful integration of global surveillance data, African epidemiology, DRC subnational burden, and travel-associated spread. The manuscript is generally well written, methodologically rigorous, and timely. The public health message—that emergency termination does not indicate elimination of disease transmission—is important and clearly conveyed.

The manuscript is valuable and clinically relevant. However, several areas would benefit from clarification or expansion to improve interpretability, strengthen clinical relevance, and avoid potential over-interpretation of surveillance findings.

**Major Comments**

**1. Greater clinical context regarding clade-specific disease characteristics is needed**

The manuscript appropriately distinguishes clade Ia, Ib, and IIb epidemiology. However, the discussion remains predominantly surveillance-focused and provides limited clinical context for readers from infectious diseases or clinical medicine.

Given the increasing clinical interest in clade Ib, the manuscript would benefit from a brief section discussing known or suspected differences between clades regarding: Clinical severity, Transmission dynamics, Population groups affected, Mortality patterns and Role of sexual versus non-sexual transmission

The statement regarding lower CFR in eastern DRC being “compatible with clade Ib predominance” is appropriately cautious, but more context is needed to avoid readers interpreting this as evidence of lower virulence.

**Questions for the authors:**

1a. Can the authors briefly summarize current evidence regarding clinical differences between clade Ib and previous clades?

1b. Are there available clinical datasets that support or contradict differences in severity between clades?

1c. Could differing age distributions or healthcare access explain some of the CFR observations more than viral lineage?

---

**2. Interpretation of case fatality ratio (CFR) requires stronger caution**

Although limitations are acknowledged, CFR interpretation throughout the manuscript should be more conservative. CFR comparisons based on surveillance data are particularly vulnerable to: Underdiagnosis of mild disease, Variable testing availability, Differences in healthcare infrastructure, Reporting completeness, Delayed mortality reporting.

This issue is especially important when comparing eastern versus non-eastern DRC provinces and different WHO regions.

For example, lower CFR in eastern provinces may reflect: More active surveillance due to outbreak attention, Earlier diagnosis, Greater ascertainment of mild cases, Younger affected populations and Differential healthcare access and referral systems

**Questions for the authors:**

2a. Do the authors have age-adjusted or severity-adjusted regional data?

2b. Were hospitalization or severe disease indicators available at regional level?

2c. Can the authors expand discussion to better emphasize the limitations of CFR comparisons using aggregated surveillance data?

---

**3. Community transmission terminology should be clarified**

The definition of community transmission (CT) is provided in the methods; however, from a clinical and infection prevention perspective, the term may be interpreted differently by readers.

Currently, CT is defined as “any documented onward transmission after importation.” This may unintentionally imply sustained local outbreaks when, in reality, some events may represent only limited secondary spread.

A clearer distinction between:

single onward transmission events, and sustained community transmission would improve clarity and reduce misinterpretation.

**Questions for the authors:**

3a. Were authors able to distinguish isolated secondary cases from sustained local transmission?

3b. Could alternative wording such as “documented secondary transmission” be considered?

---

**4. Interpretation of travel-linked pathways should be further clarified**

The observation that the UAE appeared frequently as a travel-linked transit point is epidemiologically interesting and likely reflects major international travel connectivity. However, travel history may not necessarily represent the country of acquisition or transmission, particularly in highly connected transit settings.

The manuscript appropriately notes this limitation, but I suggest emphasizing this point further in the Discussion to avoid over-interpretation of travel-linked pathways as evidence of epidemiological amplification or local transmission risk without supporting exposure or genomic data.

**Questions for the authors:**

4a. Were travel pathways derived solely from reported travel history, or were exposure investigations incorporated when available?

4b. Was any genomic epidemiology available to support inferred travel-associated linkage between countries?

4c. Could major aviation hubs or transit-country effects have influenced the observed frequency of reported linkage, particularly in settings with high international passenger movement?

---

**5. The manuscript occasionally risks implying causal relationships from temporal associations**

The authors appropriately caution against causal interpretation, which is appreciated. However, several sections may still be interpreted as implying policy interventions directly influenced epidemic trajectories.

For example: PHEIC declaration, April 2025 SPRP update, Financing disruption analyses

These events coincide with changing epidemic phases, but causality cannot be inferred from interrupted time-series alone.

The manuscript would benefit from even more cautious wording in selected sections.

**Questions for the authors:**

5a. Could the authors clarify whether sensitivity analyses were performed using alternative breakpoints?

5b. Were lag periods considered for policy-response effects?

5c. Could observed changes simply reflect the natural epidemic curve?

---

**6. More discussion on surveillance bias would strengthen interpretation**

One major concern is that changes in case counts may reflect surveillance intensity rather than true incidence. Periods immediately after PHEIC declaration may have included: Increased testing, Expanded case definitions, Greater international reporting, Increased clinician awareness

This issue is mentioned, but could be expanded further because it substantially affects interpretation of reported incidence rate ratios.

**Questions for the authors**

6a. Were testing volumes or positivity rates available?

6b. Did any countries undergo major surveillance changes during the study period?

6c. Can the authors discuss how surveillance intensity may have influenced apparent epidemic growth?

---

**7. More clinical interpretation of the demographic findings would be valuable**

The demographic section is informative but somewhat detached from clinical interpretation. The changing proportions of MSM status, travel history, and sexual transmission are important observations and deserve more contextual discussion. For example: Do these findings suggest increasing concentration in sexual networks outside Africa? Could reporting differences explain some of the trends? How should clinicians interpret these shifts in exposure patterns?

**Questions for the authors:**

7a. Can the authors better contextualize the observed rise in MSM-associated and travel-associated reporting?

7b. Could missing data or reporting bias partly explain these findings?

---

**Minor Comments**

1. The manuscript is generally well written, though several sections in the Results and Discussion are dense and statistically heavy. Shortening some long sentences may improve readability for clinicians.

2. Consider adding a brief schematic or summary figure showing:

- major epidemic phases,

- clade predominance,

- geographic spread,

- major policy milestones.

3. Please define abbreviations at first use consistently (e.g., CT, SPRP, PHECS).

4. The DRC hotspot classification methodology is interesting but may benefit from a short explanation in simpler terms for non-epidemiology readers.

5. Consider briefly discussing implications for clinicians outside endemic regions, particularly regarding travel-associated diagnosis and imported cases.

**Final Recommendation**

This is a strong and timely manuscript with clear public health importance. The work is comprehensive and methodologically sound. My comments are mainly directed toward improving clinical interpretability, strengthening caution around surveillance-based inference, and enhancing clarity for infectious disease and frontline clinical readers. I believe the manuscript would be strengthened substantially after minor-to-moderate revision.
